# Pharmacokinetic–pharmacodynamic modeling of maintenance therapy for childhood acute lymphoblastic leukemia

**DOI:** 10.1038/s41598-023-38414-0

**Published:** 2023-07-20

**Authors:** Anna Gebhard, Patrick Lilienthal, Markus Metzler, Manfred Rauh, Sebastian Sager, Kjeld Schmiegelow, Linea Natalie Toksvang, Jakob Zierk

**Affiliations:** 1 MathOpt group, Institute of Mathematical Optimization, Faculty of Mathematics, Otto von Guericke University Magdeburg, Magdeburg, Germany; 2 Department of Pediatrics and Adolescent Medicine, University Hospital Erlangen, Erlangen, Germany; 3 Department of Pediatrics and Adolescent Medicine, University Hospital Rigshospitalet, Copenhagen, Denmark; 4 Institute of Clinical Medicine, Faculty of Medicine, University of Copenhagen, Copenhagen, Denmark

**Keywords:** Computational biology and bioinformatics, Oncology

## Abstract

In the treatment of childhood acute lymphoblastic leukemia (ALL), current protocols combine initial high-dose multiagent chemotherapy with prolonged oral therapy with 6-mercaptopurine (6MP) and low-dose methotrexate (MTX) maintenance therapy. Decades of research on ALL treatment have resulted in survival rates of approximately 90%. However, dose-response relationships vary widely between patients and insight into the influencing factors, that would allow for improved personalized treatment management, is insufficient. We use a detailed data set with measurements of thioguanine nucleotides and MTX in red blood cells and absolute neutrophil count (ANC) to develop pharmacokinetic models for 6MP and MTX, as well as a pharmacokinetic–pharmacodynamic (PKPD) model capable of predicting individual ANC levels and thus contributing to the development of personalized treatment strategies. Here, we show that integrating metabolite measurements in red blood cells into the full PKPD model improves results when less data is available, but that model predictions are comparable to those of a fixed pharmacokinetic model when data availability is not limited, providing further evidence of the quality of existing models. With this comprehensive model development leading to dynamics similar to simpler models, we validate the suitability of this model structure and provide a foundation for further exploration of maintenance therapy strategies through simulation and optimization.

## Introduction

Acute lymphoblastic leukemia (ALL) is the most common pediatric malignancy and is characterized by the pathological proliferation of malignant lymphoblasts with consecutive displacement of the normal hematopoiesis^1, 2^. Combination chemotherapy is the mainstay of treatment, and consists of induction, consolidation, and reinduction cycles, which are followed by oral maintenance therapy with low-dose methotrexate (MTX) and 6-mercaptopurine (6MP) until two years after start of treatment. During maintenance therapy, the doses of MTX and 6MP are adjusted according to patients’ blood counts, including the white blood cell count (WBC) or the absolute neutrophil count (ANC)^3, 4^. Even though survival rates of ALL have reached about 90%^1, 2^, understanding the pharmacokinetics (PK) and pharmacodynamics (PD) during maintenance therapy remains an unmet challenge. One promising approach is deriving a comprehensive mathematical model and predicting the effect of MTX and 6MP on individual patients as well as personalizing treatment schedules during maintenance therapy. This is especially important as WBC shows natural variation between patients, indicating a need for individualized target ranges for maintenance therapy^3, 4^.

Developing models for treatment personalization in oncology by using mathematical modeling is an increasingly important approach^5–7^, and in the field of leukemia treatment, several models have already been constructed^8^, including the models in Le et al., Jost et al. and Jayachandran et al. in terms of ALL treatment^9–11^. But the literature still lacks a model that is able to adjust the pharmacokinetics to individual patient measurements, which is extremely relevant as both MTX and 6MP display large interindividual pharmacokinetic variability^3, 12–14^, resulting in widely different concentrations. We build on the model of Jost et al.^10^ to fill this gap by using a data set that does not only include observations of the ANC, but also of thioguanine nucleotides (E-TGN) and methotrexate (E-MTX) in the red blood cells. While the model of Jost et al.^10^ builds on ANC observations only and therefore does not estimate the pharmacokinetic parameters, but fixes them population wide, the more detailed data set used here allows for the additional estimation of individual pharmacokinetic parameters describing the drug concentration in the red blood cells. Our aim is to investigate if this can provide more accurate predictions for individual patients and to what extent the treatment dynamics are already modeled in sufficient detail.

Using the aforementioned data set, we first develop and analyze solely pharmacokinetic models for MTX and 6MP which are able to predict the concentrations of E-MTX and E-TGN for individual patients. Following Jost et al.^10^, these models are then used in combination with the Friberg et al.^15^ model to construct a full pharmacokinetic–pharmacodynamic (PKPD) model replicating the effect of low-dose MTX and 6MP on ANC during maintenance therapy. This model is then compared to an estimation with the model by Jost et al.^10^, identifying scenarios where differences in model prediction accuracy occur.

## Patients and methods

### Data

The data used for modeling is a subset of the data set described in Schmiegelow et al.^16^, which was part of the Nordic Society for Paediatric Haematology and Oncology (NOPHO) ALL-92 study^17^. The protocol was approved by the ethical committee of Copenhagen (no. V.200.2080/91) as well as by the local ethical committees, and participants gave informed consent. All methods were performed in accordance with the relevant guidelines and regulations. Patients with precursor–B-cell childhood ALL were treated according to the NOPHO ALL-92 protocol with a combination of daily oral 6MP, weekly low-dose oral MTX, oral Prednisone, intravenous Vincristine, high-dose intravenous MTX and intrathecal MTX during maintenance therapy. Observations include regular measurements of the concentration of E-TGN and E-MTX in the red blood cells (both approximately monthly), and the ANC (approximately every other week). The 6MP and low-dose MTX treatment started with dosages of 75 mg/m^2^/day (6MP) and 20 mg /m^2^/week (MTX), which were reduced (increased) in the control group if the WBC count was below (above) the target range of 1.5 to 3.5 G/L, and suspended if either the WBC was less than 1.0 G/L or the thrombocyte count was below 100 G/L. The pharmacology group’s treatment was additionally adjusted according to the product of E-TGN and E-MTX measurements, with an increase in dosage if the product of E-TGN and E-MTX fell below 1,350 (nmol/mmol hemoglobin [Hb])^2^. Our model focuses similar to Jost et al. on the ANC, leading to a target range of 0.5 to 2.0 G/L, although different ANC targets are used in other studies^10^.

Since 6MP and low-dose MTX constitute the foundation for current protocols for maintenance therapy^3, 4^, we focused on modeling the effect of both and therefore excluded the time period with high-dose intravenous MTX. Additionally, our model depends on observations of E-TGN and E-MTX as initial values. The data set of each patient thus begins with the first measurement of E-TGN and E-MTX 28 days after the last high-dose intravenous MTX treatment. Further exclusion of data occurred in the following three cases in which parameter estimation would not have been possible: i) patients with less than two E-TGN or E-MTX observations, ii) patients with no 6MP or MTX dose, and iii) patients with no height, weight, or ANC observation. After these adjustments, the data set consisted of 452 patients with 4624 E-TGN, 4192 E-MTX, and 9808 ANC observations (Table 1). In the original data, both E-MTX and E-TGN were measured in nmol/mmol Hb. For unit consistency in our model, we converted both observations to µmol/L by assuming a molecular weight for hemoglobin of 64458 g/mol and a concentration of hemoglobin in erythrocytes of 330 g/L^18^.

**Table 1:** Demographic and clinical characteristics of the patient population.

Characteristic	Median	Range (min-max)
Age (years)	5.9	2.4–16.9
Weight (kg)	21.5	10.3–105.5
Height (cm)	114.0	81.5–180.0
6MP daily dose (mg/m^2^)	57.1	5.4–175.0
MTX weekly dose (mg/m^2^)	15.0	1.3–45.0
E-TGN (µmol/L)	0.83	0–7.6
E-MTX (µmol/L)	0.026	0–0.10
ANC (G/L)	1.6	0–22.5

### Population pharmacokinetic–pharmacodynamic modeling

The overall aim of our approach is to develop a model that is able to replicate the effects of low-dose MTX and 6MP on the individual patient’s ANC. We build two PK models for MTX and 6MP that predict the concentrations of E-TGN and E-MTX in the red blood cells, which are then used as input for the effect function that models a decreasing renewal rate of proliferating cells in response to an increase in E-TGN or E-MTX. The full PKPD model is then able to predict the ANC depending on the MTX and 6MP dose. Along the lines of Schmiegelow et al.^19^ we derive the MTX and 6MP PK models to replicate the metabolism and interaction of MTX and 6MP (cf. Supplementary Figures S1–S3 online for a comparison to Figure 3 in Schmiegelow et al.^19^ and Figure 1 in Toksvang et al.^3^). An overview of all investigated model variants can be found in Supplementary Table S3 online.

**Figure 1:**
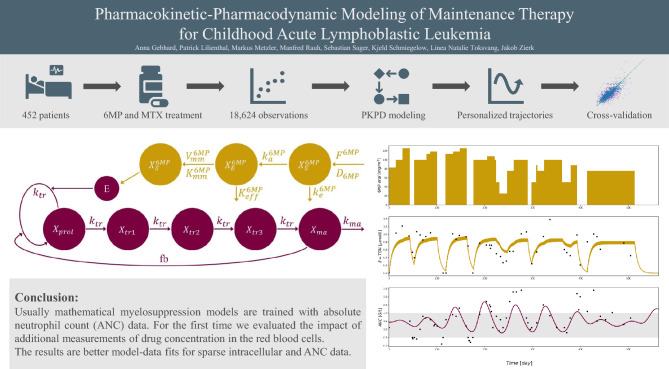
Visual abstract displaying an overview of the modeling process, the resulting model and simulated trajectories.

#### Methotrexate pharmacokinetics

In line with previously published models^9, 20–22^, our PK compartment model for MTX describes the concentration of MTX including plasma and intracellular concentrations.

Panetta et al. use the data of MTX plasma levels and MTX and methotrexate polyglutamate (MTXPG) observations in leukemia cells of 194 patients with newly diagnosed ALL to model the concentration of MTX and MTXPG in leukemia cells during the treatment of childhood ALL with high-dose MTX infusions^20, 21^. The 2-compartment model describing the plasma concentrations is complemented by two compartments modeling the intracellular kinetics with influx being described by Michaelis–Menten kinetics and the efflux by a linear elimination rate. The model by Korell et al. is based on MTXPG measurements in the red blood cells of 48 adult patients with rheumatoid arthritis treated with low-dose oral or subcutaneous MTX^22^. The parameters of the 2-compartment plasma PK model with first-order absorption were fixed for all patients, the parameter estimation was limited to a subset of those of the five compartments describing the intracellular kinetics with the influx from the plasma being modeled using a linear rate, and the elimination from the red blood cells being described by a clearance parameter. Le et al. build their model on the work of Panetta et al., while replacing the 2-compartment plasma PK model with a 1-compartment model with first-order absorption and fixed parameters^9, 20, 21^.

With these three models as a starting point, we analyzed different model variants to find the best fit for our data. The two major components we modified were the plasma PK model and the intracellular kinetics. Since our data included no plasma levels of MTX, we followed Korell et al. and Le et al. and fixed the parameters of the plasma PK model for all patients^9, 22^. The different models we tested were a 2-compartment model with first-order absorption and the parameter values based on Panetta et al. and Le et al.^9, 20, 21^, the same plasma PK model with dose-dependent bioavailability calculated according to Ogungbenro et al.^23^ and a plasma PK model based on Medellin–Garibay et al. with covariate-dependent parameter values^24^. An overview of the parameter values (Supplementary Table S1 online) and a summary of the calculation of the parameter values can be found in the supplementary information online. The intracellular kinetics were estimated as fixed effects with interindividual variability and either modeled as linear or Michaelis–Menten influx kinetics combined with a linear elimination rate. The influx of MTX into the red blood cells is typically based on the central compartment describing the plasma concentrations^9, 20–22^, but there is also evidence that the integration of MTX into the red blood cells occurs in the bone marrow^25–28^. To account for this possibility, we also included model variants with the influx of MTX into the red blood cells based on the peripheral compartment. Supplementary Figure S4 online illustrates the resulting structurally different compartment model variants with the corresponding system of ordinary differential equations (ODEs) below:

MTX1 $$\frac{d{X}_{GI}^{MTX}}{dt}= -{k}_{a}^{MTX}\cdot {X}_{GI}^{MTX}$$

MTX2 $$\frac{d{X}_{C}^{MTX}}{dt}={k}_{a}^{MTX}\cdot {X}_{GI}^{MTX}-{k}_{e}^{MTX}\cdot {X}_{C}^{MTX}-{k}_{cp}\cdot {X}_{C}^{MTX}+{k}_{pc}\cdot {X}_{P}^{MTX}$$

MTX3 $$\frac{d{X}_{P}^{MTX}}{dt}={k}_{cp}\cdot {X}_{C}^{MTX}-{k}_{pc}\cdot {X}_{P}^{MTX}$$

Linear kinetics, central compartment:

MTX4a $$\frac{d{X}_{E}^{MTX}}{dt}={K}_{in}^{MTX}\cdot \frac{{X}_{C}^{MTX}}{{V}_{C}^{MTX}}-{K}_{eff}^{MTX}\cdot {X}_{E}^{MTX}$$

Michaelis–Menten kinetics, central compartment:

MTX4b $$\frac{{dX_{E}^{MTX} }}{dt} = \frac{{V_{mm}^{MTX} \cdot {{X_{C}^{MTX} } \mathord{\left/ {\vphantom {{X_{C}^{MTX} } {V_{C}^{MTX} }}} \right. \kern-0pt} {V_{C}^{MTX} }}}}{{K_{mm}^{MTX} + {{X_{C}^{MTX} } \mathord{\left/ {\vphantom {{X_{C}^{MTX} } {V_{C}^{MTX} }}} \right. \kern-0pt} {V_{C}^{MTX} }}}} - K_{eff}^{MTX} \cdot X_{E}^{MTX}$$

Linear kinetics, peripheral compartment:

MTX4c $$\frac{d{X}_{E}^{MTX}}{dt}={K}_{in}^{MTX}\cdot \frac{{X}_{P}^{MTX}}{{V}_{P}^{MTX}}-{K}_{eff}^{MTX}\cdot {X}_{E}^{MTX}$$

Michaelis–Menten kinetics, peripheral compartment:

MTX4d $$\frac{{dX_{E}^{MTX} }}{dt} = \frac{{V_{mm}^{MTX} \cdot {{X_{P}^{MTX} } \mathord{\left/ {\vphantom {{X_{C}^{MTX} } {V_{P}^{MTX} }}} \right. \kern-0pt} {V_{P}^{MTX} }}}}{{K_{mm}^{MTX} + {{X_{P}^{MTX} } \mathord{\left/ {\vphantom {{X_{P}^{MTX} } {V_{P}^{MTX} }}} \right. \kern-0pt} {V_{P}^{MTX} }}}} - K_{eff}^{MTX} \cdot X_{E}^{MTX}$$

Following previously published models, we assumed the influx and efflux of MTX into and out of the red blood cells to not have any noticeable effect on the concentration in the central compartment^20–22^. It was not possible to initialize all compartments with 0 as the data set for estimations includes only observations after the onset of maintenance therapy and MTX is accumulated in the red blood cells. We therefore initialized the compartments in the following way:

$${X}_{GI}^{MTX}\left(0\right)=0$$

$${X}_{C}^{MTX}\left(0\right)=0$$

$${X}_{P}^{MTX}\left(0\right)=0$$

$${X}_{E}^{MTX}\left(0\right)=INIMTX$$

with INIMTX being the first observation in the data set at time point 0. For the most promising MTX PK model variant, we also tested an estimation of the initial value with ${X}_{E}^{MTX}(0)$ modeled as a patient-specific parameter, and the possibility to let ${X}_{E}^{MTX}(0)$ vary according to the residual error model with ${X}_{E}^{MTX}\left(0\right)= (INIMTX- {\sigma }_{2}^{MTX}\cdot {\eta }_{2}^{INIMTX})/(1+ {\sigma }_{1}^{MTX}\cdot {\eta }_{1}^{INIMTX})$.

#### 6-mercaptopurine pharmacokinetics

The starting point for constructing the 6MP pharmacokinetic model were the previously published models by Hawwa et al. and Jayachandran et al.^11, 29^ Hawwa et al. use data from 19 children with ALL receiving 6MP during maintenance therapy to build a 3-compartment model with one compartment and first-order absorption describing the plasma levels of 6MP as well as two compartments modeling the intracellular 6MP metabolites 6-thioguanine nucleotides and 6-methylmercaptopurine nucleotides using linear kinetics^29^. The parameters for the plasma PK model were fixed to values from the literature, while the parameters for the intracellular dynamics were estimated using NONMEM. Jayachandran et al. use the same structural model for their 6MP PK model as Hawwa et al., but describe the influx in the red blood cells by Michaelis–Menten kinetics^11, 29^. Data from the literature was used for estimation and most parameters were fixed to values from the literature, including Hawwa et al.^29^.

We follow both models in describing the plasma kinetics of 6MP with a 1-compartment model with first-order absorption and fixed parameter values but use mainly the results of Lennard et al. for calculating the parameters^30^. Even though Lennard et al. perform a non-compartmental analysis of the data of 19 patients with childhood ALL during maintenance therapy^30^, it is possible to determine the parameters of a 1-compartment model with the resulting values. Supplementary Table S2 online shows an overview of the parameters, the calculation is detailed in the supplementary information online. Similar to the MTX PK model, the intracellular kinetics of 6MP were estimated as fixed effects with interindividual intervariability and either modeled as linear or Michaelis–Menten influx kinetics combined with a linear elimination rate, resulting in the model variants $P{K}_{mm}^{6MP}$ and $P{K}_{lin}^{6MP}$. Both compartment models are visualized in Supplementary Figure S5 online and are described by the following ODE system:

6MP1 $$\frac{d{X}_{GI}^{6MP}}{dt}= -{k}_{a}^{6MP}\cdot {X}_{GI}^{6MP}$$

6MP2 $$\frac{d{X}_{GI}^{6MP}}{dt}= {k}_{a}^{6MP}\cdot {X}_{GI}^{6MP}-{k}_{e}^{6MP}\cdot {X}_{C}^{6MP}$$

Linear kinetics:

6MP3a $$\frac{d{X}_{E}^{6MP}}{dt}={K}_{in}^{6MP}\cdot \frac{{X}_{C}^{6MP}}{{V}_{C}^{6MP}}-{K}_{eff}^{6MP}\cdot {X}_{E}^{6MP}$$

Michaelis–Menten kinetics:

6MP3b $$\frac{{dX_{E}^{6MP} }}{dt} = \frac{{V_{mm}^{6MP} \cdot {{X_{C}^{6MP} } \mathord{\left/ {\vphantom {{X_{C}^{6MP} } {V_{C}^{6MP} }}} \right. \kern-0pt} {V_{C}^{6MP} }}}}{{K_{mm}^{6MP} + {{X_{C}^{6MP} } \mathord{\left/ {\vphantom {{X_{C}^{6MP} } {V_{C}^{6MP} }}} \right. \kern-0pt} {V_{C}^{6MP} }}}} - K_{eff}^{6MP} \cdot X_{E}^{6MP}$$

We again assume the influx and efflux of 6MP into and out of the red blood cells to not influence plasma kinetics and initialize the compartments similar to the MTX PK model with the first observation INITGN in the following way:

$${X}_{GI}^{6MP}\left(0\right)=0$$

$${X}_{C}^{6MP}\left(0\right)=0$$

$${X}_{E}^{6MP}\left(0\right)=INITGN$$

For the most promising 6MP PK model variant, we again also tested an estimation of the initial value with ${X}_{E}^{6MP}(0)$ modeled as a patient-specific parameter, and the possibility to let ${X}_{E}^{6MP}(0)$ vary according to the residual error model with ${X}_{E}^{6MP}\left(0\right)= (INITGN- {\sigma }_{2}^{6MP}\cdot {\eta }_{2}^{INITGN})/(1+ {\sigma }_{1}^{6MP}\cdot {\eta }_{1}^{INITGN})$.

#### Pharmacodynamics

The full PKPD model was then built combining the two PK models with the state-of-the-art myelosuppression model by Friberg et al.^15^ following Le et al. and Jost et al.^9, 10^ The additional part of the model consists of five compartments with one compartment describing the proliferating cells, three transit compartments, and one compartment modeling the mature cells circulating in the blood (cf. Supplementary Figure S6 online, where this part of the PKPD model is visualized). The PK and the PD parts of the model are linked by a linear effect function which models the effect of the concentrations of MTX and TGN in the red blood cells on the proliferating rate. We tested different effect functions based on either E-TGN alone or on E-TGN and E-MTX combined. The PD part of the model is described by the ODE system below:

PKPD1 $$\frac{d{X}_{prol}}{dt}={k}_{tr}\cdot {X}_{prol}\cdot \left(1-{E}_{drug}\right)\cdot fb-{k}_{tr}\cdot {X}_{prol}$$

PKPD2 $$\frac{d{X}_{tr1}}{dt}={k}_{tr}\cdot ({X}_{prol}-{X}_{tr1})$$

PKPD3 $$\frac{d{X}_{tr2}}{dt}={k}_{tr}\cdot ({X}_{tr1}-{X}_{tr2})$$

PKPD4 $$\frac{d{X}_{tr3}}{dt}={k}_{tr}\cdot ({X}_{tr2}-{X}_{tr3})$$

PKPD5 $$\frac{d{X}_{ma}}{dt}={k}_{tr}\cdot {X}_{tr3}-{k}_{circ}\cdot {X}_{ma}$$

with

PKPD6 $$fb= {\left(\frac{base}{{X}_{ma}}\right)}^{\gamma }$$

Effect function based on E-TGN:

PKPD7a $${E}_{drug}=slop{e}^{6MP}\cdot {X}_{E}^{6MP}$$

Effect function based on E-TGN and E-MTX:

PKPD7b $${E}_{drug}=slop{e}^{6MP}\cdot {X}_{E}^{6MP}+slop{e}^{MTX}\cdot {X}_{E}^{MTX}$$

Similar to Jost et al.^10^ we fixed both the proliferating rate of the first compartment and ${k}_{circ}$, with ${k}_{prol}= {k}_{tr}$ and ${k}_{circ}$ = 2.3765 1/day. Due to the data set beginning after weeks of treatment during maintenance therapy, we cannot assume ${X}_{ma}(0)=base$ as an initial state. Instead, we assume to have reached a treatment steady state with an additional parameter inieff describing the drug effect at the initial time point and ${X}_{ma}(0)=inieff \cdot base$, leading to the initial states below.

$${X}_{prol}\left(0\right)=inieff \cdot base \cdot \frac{{k}_{ma}}{{k}_{tr}}$$

$${X}_{tr1}\left(0\right)=inieff \cdot base \cdot \frac{{k}_{ma}}{{k}_{tr}}$$

$${X}_{tr2}\left(0\right)=inieff \cdot base \cdot \frac{{k}_{ma}}{{k}_{tr}}$$

$${X}_{tr3}\left(0\right)=inieff \cdot base \cdot \frac{{k}_{ma}}{{k}_{tr}}$$

$${X}_{ma}\left(0\right)=inieff \cdot base$$

Additionally to the resulting model variants, we also estimated the model of Jost et al. with our data set to be able to compare the results of integrating individual E-TGN and E-MTX measurements in the model developing process in contrast to a fully fixed pharmacokinetic submodel^10^.

#### Interindividual Variability and residual error model

Using the nonlinear mixed effects (NLME) approach for parameter estimation, we assumed a log-normal distribution for the interindividual variability of all models as is conventional in the literature leading to the following description of the patient-specific parameters ${\uptheta }_{i}^{k}$ with $\theta$ = (${\uptheta }^{k}$) as the vector of fixed effect parameters, k in {${K}_{in}^{MTX}$, ${V}_{mm}^{MTX}$, ${K}_{mm}^{MTX}$, ${K}_{eff}^{MTX}$, ${K}_{in}^{6MP}$, ${V}_{mm}^{6MP}$, ${K}_{mm}^{6MP}$, ${K}_{eff}^{6MP}$, $base$, ${k}_{tr}$, $slop{e}^{6MP}$, $slop{e}^{MTX}$, γ, $inieff$} and i=1,…,n as the patient indices:

$${\theta }_{i}^{k}= {\theta }^{k}\cdot \mathrm{exp}({\eta }_{i}^{k})$$

with each ${\eta }^{k}$ following a normal distribution $N(0, {\omega }_{k}^{2})$. After obtaining the first estimation results of the PK models, we added two model variants $P{K}_{fix,mm,cent,pop}^{MTX}$ and $P{K}_{mm,pop}^{6MP}$ where ${K}_{mm,i}^{MTX}={K}_{mm}^{MTX}$ and ${K}_{mm,i}^{6MP}={K}_{mm}^{6MP}$ are not associated with any interindividual variability, see Section "Results" for further discussion.

We tested different residual error models for both PK models with the best performing being a combined additive-proportional model:

$${Y}_{ij}^{PK}={X}_{E}^{PK}\left({t}_{ij}\right)+{X}_{E}^{PK}\left({t}_{ij}\right)\cdot {\epsilon }_{ij}^{PK,1}+ {\epsilon }_{ij}^{PK,2}$$

with i=1,…,n as the patient indices, j=1,…,${m}_{i}^{PK}$ as the patient-specific observations, and the error terms ${\epsilon }^{PK,1} \sim N(0, {\sigma }_{PK,1}^{2})$ and ${\epsilon }^{PK,2} \sim N(0, {\sigma }_{PK,2}^{2})$ normally distributed. We compared the results of the most promising PK model variants with changing the residual error model to a purely additive (${Y}_{ij}^{PK}={X}_{E}^{PK}\left({t}_{ij}\right)+ {\epsilon }_{ij}^{PK}$) or a purely proportional residual error model (${Y}_{ij}^{PK}={X}_{E}^{PK}\left({t}_{ij}\right)+ {X}_{E}^{PK}\left({t}_{ij}\right)\cdot {\epsilon }_{ij}^{PK}$).

For the PD model, we used a proportional residual error model as this gives more weight to small values of the ANC observations, which are particularly important for treatment outcome:

$${Y}_{ij}^{ANC}={X}_{ma}\left({t}_{ij}\right)+{X}_{ma}\left({t}_{ij}\right)\cdot {\epsilon }_{ij}^{ANC}$$

with i=1,…,n as the patient indices, j=1,…,${m}_{i}^{ANC}$ as the patient-specific observations, and the error term ${\epsilon }^{ANC}\sim N(0,{\sigma }_{ANC}^{2})$ normally distributed.

### Cross-validation

The cross-validation data set was constructed by determining the time point 50% of the ANC observations lie before per patient and using this as a cut off point for E-MTX, E-TGN and ANC observations. Patients were excluded from this data set for the same reasons as described in Section "Data".

### Sensitivity analysis

A sensitivity analysis of the final PKPD model was conducted for all fixed parameters by determining the 95% confidence intervals of the absorption rate ${\mathrm{k}}_{\mathrm{a}}^{6\mathrm{MP}}$ = 21.07, the elimination rate ${k}_{e}^{6MP}$ = 15.40 and the central volume ${\mathrm{V}}_{\mathrm{C}}^{6\mathrm{MP}}$, the interval from 50% to 150% of the bioavailability F=0.12, as no information on the standard error was available, and the 95% confidence interval of ${t}_{0.5}$= 0.29 and computing the death rate of mature neutrophils ${k}_{circ}$ = ln(2)/${t}_{0.5}$ correspondingly. After that, 100 simulations with the disturbed parameters equally spaced in the interval were performed. One of the fixed parameters was varied in each simulation round, while all other fixed parameters were left at their original value and all estimated parameters were fixed to the population parameter values. The resulting ANC trajectories were then compared to the reference ANC trajectory simulated with the original values of the fixed parameters by computing the absolute distance at each time step.

### Software

The software used for parameter estimation was NONMEM 7.5, the PK models were estimated with first-order conditional estimation with interaction (FOCEi), the full PKPD model with a combination of Monte Carlo Importance Sampling Expectation Maximization (IMP) and Stochastic Approximation Expectation Maximization (SAEM). Perl speaks NONMEM (PsN) was used to create the visual predictive checks^31^. Model analysis and simulation was done with Python 3.10 using CasADI and CVODES^32, 33^.

## Results

### Methotrexate Pharmacokinetics

We compared 20 different MTX PK models to find the most suitable model, the overview of the parameter estimation can be found in Supplementary Table S4 online. There, the objective function value, RMSEs and MAEs of the individual predictions of all model variants are presented and evaluated in the same section of the supplementary information online. Analysing the results led to two structurally different model variants as best candidates for the MTX PK model, namely $P{K}_{fix,bio,lin,peri}^{MTX}$ with linear kinetics and $P{K}_{fix,mm,cent,pop}^{MTX}$ with Michaelis–Menten kinetics.

Comparing the cross-validation of both, $P{K}_{fix,bio,lin,peri}^{MTX}$ leads to more robust results, with not only the RMSEs and MAEs again increasing only slightly, but also the fixed effect parameters – in contrast to model $P{K}_{fix,mm,cent,pop}^{MTX}$ – displaying almost no change, as is reported in Table 2. The comparison of the individual patient parameters shows good agreement (cf. Supplementary Figure S8 online), too. Details of the comparison can be found in the supplementary information online.

**Table 2:** Parameter values of the model ${\mathrm{PK}}_{\mathrm{fix},\mathrm{bio},\mathrm{lin},\mathrm{peri}}^{\mathrm{MTX}}$ estimated with the whole and the cross-validation data set, relative standard error (RSE) of fixed effect parameters and residual unexplained variability in brackets.

	${PK}_{fix,bio,lin,peri}^{MTX}$	${PK}_{fix,bio,lin,peri}^{MTX}$, cross-validation
Fixed effects parameters with RSE in %		
${K}_{in}^{MTX}$[1/day]	0.031 (5)	0.030 (6)
${K}_{eff}^{MTX}$[1/day]	0.018 (5)	0.019 (5)
CV with 95% CI, η-Shrinkage in % and *p*-values		
${K}_{in}^{MTX}$	34 [29, 38], 29, 0.22	37 [32, 42], 32, 0.12
${K}_{eff}^{MTX}$	31 [26,35], 32, 0.45	21 [14, 27], 53, 0.24
Residual unexplained variability		
Additive residual error	0.000018 (7)	0.000021 (15)
Proportional residual error	0.024 (8)	0.0068 (56)
Errors of the individual predictions		
Median of RMSEs	0.0042 (0.0028)	0.0054 (0.0045)
Mean of RMSEs	0.0047 (0.0028)	0.0064 (0.0045)
Median of MAEs	0.0033 (0.0022)	0.0041 (0.0035)
Mean of MAEs	0.0036 (0.0022)	0.0049 (0.0035)

RMSEs and MAEs of the patient trajectories computed with the individual patient parameters are each calculated for the whole data set with the standard deviation in brackets. Coefficients of variation (CV) are calculated as $\sqrt{\mathrm{exp}\left({\upomega }^{2}\right)-1}$ with ${\upomega }^{2}$ as the variance of the interindividual variability estimated by NONMEM, and the 95% CI being calculated accordingly.

Taking all results into account, even though the model $P{K}_{fix,mm,cent,pop}^{MTX}$ led to the lowest objective function value, the model $P{K}_{fix,bio,lin,peri}^{MTX}$ appears to be more robust when estimating with fewer observations and seems to work well enough in the low-dose MTX regime. This is entirely unsurprising, as Michaelis–Menten kinetics generally behave similar to linear kinetics for low concentrations. Additionally, when comparing the individual patient trajectories of both models, almost no differences are visible (cf. Supplementary Figure S9 online).

Supplementary Figure S10 online depicts the goodness-of-fit plot of the final model variant $P{K}_{fix,bio,lin,peri}^{MTX}$ for the cross-validation and Supplementary Figure S11 online for the estimation using the whole data set, both showing a good agreement of observed with predicted E-MTX values. Supplementary Figure S12 online displays the visual predictive check for this model with 1000 simulations with the final parameter values, both the median and the 2.5th percentile leading to similar results for the observed and predicted E-MTX values, while the 97.5th percentile of the observations lies most of the time slightly below the 95% CI of the predictions.

### 6-mercaptopurine pharmacokinetics

We compared two structurally different models for the 6MP pharmacokinetics: Michaelis–Menten and linear influx kinetics, resulting in the model variants $P{K}_{mm,pop}^{6MP}$ and $P{K}_{lin}^{6MP}$ as potential 6MP PK models (cf. Supplementary Table S6 online and the corresponding section in the supplementary information online for details of the evaluation).Table 3 shows the parameter values of the cross-validation for the model $P{K}_{mm,pop}^{6MP}$ and the RMSEs and MAEs of the individual predictions of both the estimation using the whole data set and the cross-validation. The RMSEs and MAEs did increase only slightly, but ${K}_{mm}^{6MP}$ changed similarly to ${K}_{mm}^{MTX}$, with its value decreasing one order of magnitude and its relative standard error reaching 206%. ${V}_{mm}^{6MP}$ and ${K}_{eff}^{6MP}$ showed only minor changes (cf. also Supplementary Figure S14 online, and the same section for a comparison to the cross-validation of the model variant $P{K}_{lin}^{6MP}$).

**Table 3:** Parameter values of the model ${\mathrm{PK}}_{\mathrm{mm},\mathrm{pop}}^{6\mathrm{MP}}$ estimated with the whole and the cross-validation data set, relative standard error (RSE) of fixed effect parameters and residual unexplained variability in brackets.

	${PK}_{mm,pop}^{6MP}$	${PK}_{mm,pop}^{6MP}$, cross-validation
Fixed effects parameters with RSE in %		
${V}_{mm}^{6MP}$[µmol/L/day]	0.096 (8)	0.067 (29)
${K}_{mm}^{6MP}$[µmol/L]	0.016 (10)	0.0021 (206)
${K}_{eff}^{6MP}$[1/day]	0.041 (8)	0.039 (10)
CV with 95% CI, η-Shrinkage in % and *p*-values		
${V}_{mm}^{6MP}$	52 [44, 59], 26, 0.38	40 [33, 47], 36, 0.18
${K}_{eff}^{6MP}$	50 [42, 57], 30, 0.63	40 [34, 46], 38, 0.43
Residual unexplained variability		
Additive residual error	0.023 (9)	0.021 (10)
Proportional residual error	0.064 (5)	0.059 (7)
Errors of the individual predictions		
Median of RMSEs	0.21 (0.16)	0.26 (0.26)
Mean of RMSEs	0.24 (0.16)	0.32 (0.26)
Median of MAEs	0.17 (0.13)	0.19 (0.21)
Mean of MAEs	0.19 (0.13)	0.24 (0.20)

RMSEs and MAEs of the patient trajectories computed with the individual patient parameters are each calculated for the whole data set with the standard deviation in brackets. Coefficients of variation (CV) are calculated as $\sqrt{\mathrm{exp}({\upomega }^{2})-1}$ with ${\upomega }^{2}$ as the variance of the interindividual variability estimated by NONMEM, and the 95% CI being calculated accordingly.

Taking all results into account, the case is not as clear as for the MTX pharmacokinetics. Even though the model with linear kinetics seems to be more robust against estimating with fewer observations, the changes in the parameter values for the model with Michaelis–Menten kinetics are not as drastic. Additionally, both the mean of the $\eta$ s differing significantly from 0 and the individual patient trajectories seem to suggest that Michaelis–Menten kinetics are necessary to characterize E-TGN dynamics. Furthermore, the goodness-of-fit plots of observed versus predicted values and the visual predictive checks prefer model $P{K}_{mm,pop}^{6MP}$. But even though model $P{K}_{mm,pop}^{6MP}$ led to overall better results, the decrease in ${K}_{mm}^{6MP}$ and its huge relative standard error led us to include model $P{K}_{lin}^{6MP}$ in the PKPD modeling to compare results again when estimating the full model.

### Pharmacodynamics

The objective function values and RMSEs and MAEs based on the individual predictions of all submodels of the five compared PKPD models can be found in Supplementary Table S8 online. Thorough evaluation of the results led to $PKP{D}_{lin,mm}^{6MP}$ as the best performing model variant with Michaelis–Menten kinetics in the 6MP PK submodel and the effect function based solely on E-TGN (cf. the supplementary information online for details of the model comparison).

The results of this model variant are mostly similar to the $PKP{D}^{Jost}$ model, but distinct differences occur when computing the RSMEs and MAEs of the population predictions of the ANC. The errors of the $PKP{D}^{Jost}$ model are almost twice as high (cf. Table 4 for a comparison with model $PKP{D}_{lin,mm}^{6MP}$ ), indicating that with no prior knowledge about the individual patient, the model estimated with integrating E-TGN observations performs better than the $PKP{D}^{Jost}$ model, which relies on values from the literature.

**Table 4:** ANC errors of the population predictions of the estimation using the whole data set, and of the individual predictions of the cross-validation of models ${\mathrm{PKPD}}^{\mathrm{Jost}}$ and ${\mathrm{PKPD}}_{\mathrm{lin},\mathrm{mm}}^{6\mathrm{MP}}$.

	${PKPD}^{Jost}$	${PKPD}_{lin,mm}^{6MP}$
ANC errors of the population predictions		
Median of RMSEs	2.12 (0.79)	1.12 (0.61)
Mean of RMSEs	2.23 (0.79)	1.27 (0.61)
Median of MAEs	1.87 (0.65)	0.90 (0.39)
Mean of MAEs	1.91 (0.65)	0.96 (0.39)
ANC errors of the individual predictions for the cross-validation		
Median of RMSEs	1.03 (2.31)	1.07 (1.01)
Mean of RMSEs	1.38 (2.31)	1.29 (1.01)
Median of MAEs	0.80 (1.40)	0.79 (0.60)
Mean of MAEs	1.00 (1.40)	0.94 (0.60)

RMSEs and MAEs are each calculated for the whole data set with the standard deviation in brackets.

The comparison of the RMSEs and MAEs of the individual predictions of the cross-validation of the model variant $PKP{D}_{lin,mm}^{6MP}$ revealed similar medians and means for all three models, but differing results for the standard deviations of the ANC errors (cf. Table 4 for the ANC errors and Supplementary Table S9 online for the E-MTX and E-TGN errors): the one of the model $PKP{D}^{Jost}$ is about 2 times higher than the one of the model variant $PKP{D}_{lin,mm}^{6MP},$ indicating more extreme deviations of the predicted ANC values from the observed ANC values. When comparing the results to those of the cross-validations of the PK submodels on their own, the RMSEs and MAEs show only negligible differences.

Table 5 shows the results of the parameter estimation using the whole data set and for the cross-validation for the model $PKP{D}_{lin,mm}^{6MP}$. The fixed effect parameter ${K}_{mm}^{6MP}$ still decreases, but not as immensely as when estimating solely the 6MP PK model. The sharp increase in its relative standard error does not occur for the cross-validation of the full PKPD model. The coefficients of variation show only negligible changes for the cross-validation.

**Table 5:** Parameter values of the model ${\mathrm{PKPD}}_{\mathrm{lin},\mathrm{mm}}^{6\mathrm{MP}}$ estimated with the whole and the cross-validation data set, relative standard error (RSE) of fixed effect parameters and residual unexplained variability in brackets.

	${PKPD}_{lin,mm}^{6MP}$		${PKPD}_{lin,mm}^{6MP}$, cross-validation	
	Fixed effect parameters with RSE in %	CV with 95% CI, η-Shrinkage in % and *p*-values	Fixed effect parameters with RSE in %	CV with 95% CI, η-Shrinkage in % and *p*-values
${K}_{in}^{MTX}$[1/day]	0.032 (1)	34 [29, 38], 29, 0.75	0.029 (2)	35 [30, 40], 34, 0.86
${K}_{eff}^{MTX}$[1/day]	0.019 (1)	31 [26, 35], 30, 0.77	0.018 (1)	26 [20, 31], 45, 0.72
${V}_{mm}^{6MP}$[µmol/L/day]	0.21 (4)	56 [46, 65], 27, 0.97	0.13 (4)	42 [34, 50], 35, 0.98
${K}_{mm}^{6MP}$[µmol/L]	0.14 (0.1)	-	0.046 (7)	-
${K}_{eff}^{6MP}$[1/day]	0.050 (2)	54 [45, 63], 29, 0.97	0.044 (2)	44 [37, 51], 33, 0.91
$base$[G/L]	2.17 (3)	27 [24, 31], 22, 0.68	2.08 (4)	29 [25, 32], 27, 0.78
${k}_{tr}$[1/day]	0.15 (2)	72 [60, 82], 35, 0.57	0.17 (3)	72 [60, 84], 46, 0.65
${slope}^{6MP}$[L/µmol]	0.16 (4)	81 [65, 97], 40, 0.68	0.15 (6)	93 [68, 117], 45, 0.96
γ	0.79 (5)	11[8, 12], 49, 0.89	0.82 (10)	15 [11, 18], 58, 0.54
$inieff$	0.87 (22)	54 [48, 59], 20, 0.63	0.88 (29)	48 [42, 53], 27, 0.69
Residual unexplained variability				
Additive, MTX	0.000018 (7)		0.000022 (8)	
Proportional, MTX	0.024 (8)		0.0074 (25)	
Additive, 6MP	0.024 (9)		0.020 (10)	
Proportional, 6MP	0.061 (5)		0.051 (6)	
Proportional, ANC	0.25 (2)		0.22 (3)	

Coefficients of variation (CV) are calculated as $\sqrt{\mathrm{exp}({\upomega }^{2})-1}$ with ${\upomega }^{2}$ as the variance of the interindividual variability estimated by NONMEM, and the 95% CI being calculated accordingly.

Comparing the results of the parameter estimation of the full PKPD model to those of the estimation of only the PK submodels (cf. Tables 2 and 3), there are no significant changes for the MTX PK submodel and for all coefficients of variation, while for the 6MP PK submodel, both ${V}_{mm}^{6MP}$ and ${K}_{mm}^{6MP}$ increase distinctly.

**Figure 2:**
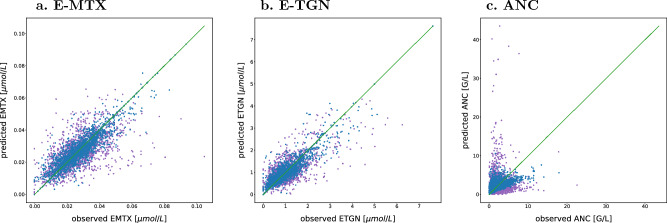
Goodness-of-fit plots of observed and by the model ${\mathrm{PKPD}}_{\mathrm{lin},\mathrm{mm}}^{6\mathrm{MP}}$ predicted E-MTX, E-TGN and ANC values. Estimations were done with the cross-validation data set, where blue dots are in-sample and purple dots are out-of-sample observations.

Figure 2 shows the goodness-of-fit plot of the final model variant $PKP{D}_{lin,mm}^{6MP}$ for the cross-validation and Supplementary Figure S20 online for the estimation using the whole data set. Both show a good agreement of observed and predicted E-MTX and E-TGN values, and more variation for the ANC values. Supplementary Figure S21 online displays the visual predictive check for this model with 1000 simulations with the final parameter values, both the median and the 97.5th percentile leading to similar results for the observed and predicted ANC values, while the 2.5th percentile of the observations lies most of the time slightly above the 95% CI of the predictions.

**Figure 3:**
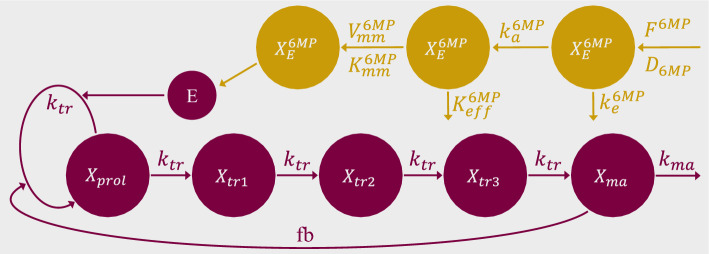
Final PKPD model with the 6MP PK submodel.

Figure 3 displays the final model structure with the 6MP PK submodel, a detailed overview of the fixed parameter values and the ODE system of the final PKPD model can be found in the supplementary information online.

### Sensitivity analysis

The sensitivity analysis of the final PKPD model led to a maximal absolute distance to the reference ANC trajectory of 0.49 for the absorption rate ${k}_{a}^{6MP}$, with smaller maximal absolute distances for all other fixed parameters, namely 0.12 for the bioavailability ${F}^{6MP}$, 0.093 for the elimination rate ${k}_{e}^{6MP}$, 0.11 for the central volume ${V}_{C}^{6MP}$ and 0.0013 for the death rate of mature neutrophils ${k}_{circ}.$ The medians and interquartile ranges of the trajectory with the maximal distance to the reference ANC trajectory can be found in Supplementary Table S12 online, plots of the results of all simulations of the sensitivity analysis in Supplementary Figures S24–28 online.

## Discussion

The mechanism of action of 6MP involves the sequential conversion to TGN and its subsequent incorporation into DNA (DNA-TG). Furthermore, methylated metabolites of 6MP generated by thiopurine methyltransferase (TPMT) directly inhibit purine de novo synthesis (PDNS), thereby increasing the incorporation of TGN into DNA. MTX works by depleting the cells of reduced folates as well as directly inhibiting PDNS, thereby working synergistically with methylated metabolites of 6MP to increase DNA-TG, which is considered their common downstream metabolite. (Supplementary Figures S1–S3)^3^ We therefore expected a rise in E-MTX and E-TGN to lead to a decrease in ANC. In the model, this is implemented by an effect function depending on either E-MTX, E-TGN or a combination of both, which reduces the proliferating rate and consequently, the number of proliferating cells, the cells in the transit compartments and ultimately, the ANC.

We compared 20 different MTX PK models. For the application addressed here, the model combining fixed plasma pharmacokinetics based on Panetta et al.^20, 21^, dose-adjusted bioavailability based on Ogungbenro et al.^23^ and linear kinetics modeling influx from the peripheral compartment into red blood cells proved to be the most appropriate. This model does not have the lowest objective function value but exhibits sufficiently good prediction of MTX dynamics in the low-dose MTX regime while being more robust to a reduction in the number of observations used for estimation than the models with Michaelis–Menten kinetics. This is in line with Korell et al.^22^ who built a model for low-dose MTX and also modeled the influx into the red blood cells by linear kinetics. While the transportation of MTX into the red blood cells is mainly a saturable process indicating the need for Michaelis–Menten kinetics^13, 34, 35^, for low concentrations, they are virtually indistinguishable from linear kinetics. Relying on linear kinetics in this case introduces the need to be especially careful though when simulating new treatment schedules to make sure to not reach MTX concentrations in the plasma where the dynamics would leave the linear phase of Michaelis–Menten kinetics, as linear kinetics are not able to describe the saturation that would occur for high MTX doses. Comparing the estimated fixed effect parameter values to those from the literature show excellent agreement for the elimination rate ${K}_{eff}^{MTX}$ = 0.018 1/day with Korell et al. reporting an elimination rate of 0.024 1/day^22^, Schalhorn et al. and Schrøder et al. reporting a half-life of 30–40 days and a mean half-life of 37 days of MTX in the red blood cells, respectively, resulting in an elimination rate of 0.017–0.023 1/day and 0.019 1/day^27, 28^. For the linear influx rate of our model, ${K}_{in}^{MTX}$ = 0.031 1/day, there are less results available in the literature with only Korell et al. reporting a much smaller rate of 0.0014 1/day^22^. The reason for this discrepancy might be the different patient population, which consists of adults with rheumatoid arthritis in contrast to the here analyzed population of children with ALL^22^.

The basis in the literature to draw from for the 6MP PK model was less comprehensive, leading to only one model for the plasma pharmacokinetics of 6MP based on the results of Lennard et al.^30^ Further comparison of the results of using linear versus Michaelis–Menten kinetics for the influx of 6MP into the red blood cells showed that for 6MP, Michaelis–Menten kinetics are necessary to adequately describe the dynamics. Still, the observations available were not sufficient to fully analyze all aspects of the Michaelis–Menten kinetics with no possibility to estimate the coefficient of variation of the parameter ${K}_{mm}^{6MP}$ and the reduced robustness against using less observations for the parameter estimation. There are several possible remedies for this: most obviously, increasing the number of observations available per patient, using a different timeline where it is possible to start the estimation at a point in time where the E-TGN compartment can be initialized with 0, and integrating observations for higher 6MP doses in the data set. Due to the limitations of our data set, it was not possible to include these remedies in our paper. The comparison of the estimated fixed effect parameter values to results from the literature is hindered by differences in units and structural models. Jayachandran et al. report a maximal influx into the red blood cells for Michaelis–Menten kinetics that is also integrated into the model of Le et al. and can be converted to about 0.23 µmol/L/day by assuming a mean corpuscular volume of 83 fL^9, 11, 36^, which is higher than the estimated ${V}_{mm}^{6MP}$ = 0.096 µmol/L/day here, but similar to that estimated using the full PKPD model. Our estimated elimination rate of ${K}_{eff}^{6MP}$ = 0.041 1/day is at the same order of magnitude as that reported by Jayachandran et al. with 0.0714 1/day^11^.

The full PKPD model was developed building on the model of Jost et al.^10^ by using the Friberg et al. model^15^, a linear effect function and either E-TGN or a combination of E-TGN and E-MTX as inputs. To further investigate the 6MP PK model, a variant with linear kinetics as well as a variant with Michaelis–Menten kinetics were integrated into the PKPD model and compared to each other. The final PKPD model relies only on E-TGN as input for the effect function and uses Michaelis–Menten kinetics for the 6MP PK model. The resulting fixed effect parameters for the 6MP PK submodel did differ from those stemming from estimating the PK model on its own, with ${V}_{mm}^{6MP}$ = 0.21 µmol/L/day and ${K}_{eff}^{6MP}$ = 0.050 1/day now being closer to the results in Jayachandran et al.^11^ Additionally, the 6MP PK submodel is more robust against estimating with less data when integrated into the PKPD model with the parameters now varying not as much and the relative standard error not increasing as much compared to estimating the 6MP PK model on its own. Focusing on the PD submodel, the parameters $base$ = 2.17 G/L, ${k}_{tr}$ = 0.15 1/day and γ = 0.79 are comparable to those reported in Jost et al. with $base$ = 2.34 G/L, ${k}_{tr}$ = 0.148 1/day and γ = 0.769^10^, which also testifies to both models replicating the same dynamics. The mean maturation time indicated by our model is comparable to that from Jost et al. with 20 days^10, 37^. The slope of the effect function of this model differs with $slop{e}^{6MP}$ = 0.16 L/µmol from the Jost et al. one^10^, which must be converted due to unit differences in the E-TGN-compartment, resulting in a slope of 0.037 L/µmol. This discrepancy might be explained by the 6MP PK dynamics in the model in Jost et al. being fixed for all patients^10^, while the observations of E-TGN in our data allowed to vary parameters individually resulting in patient-wise differing E-TGN trajectories, which influences the size of the slope parameter. This might also contribute to the improved model performance compared to $PKP{D}^{Jost}$ when predicting patient trajectories by using the population parameters. The final parameter to discuss, $inieff$ = 0.87, cannot be compared to values from the literature, as in contrast to previously published models^9, 10^, the data used for this estimation begins in the middle of maintenance therapy and the assumption of ${X}_{ma}(0)=base$ does not hold. One limitation of our approach to estimate ${X}_{ma}(0)=inieff \cdot base$ as a possibility to represent a steady treatment effect is that for some patients, the estimated $inieff$ > 1, indicating a positive effect of the treatment. This could be circumvented by using a data set that allows the initialization of the first PD compartment with ${X}_{ma}(0)=base$.

All fixed effect parameter estimates of the final PKPD model - except for $inieff$ - show relative standard errors of 5% or lower, testifying to the identifiability of the parameters. The relative standard error of $inieff$ reaches 22%, again indicating the need for a data set with observations before the start of treatment to enable the direct estimation of $base$. For the coefficients of variation, all 95% CI lie closely around the parameter estimates (cf. Table 5), and the relative standard errors of the estimates for the residual unexplained variability reach a maximum of 9%, showing parameter identifiability to be unproblematic. There is no indication of over-parameterization of the model with the η-shrinkage reported in Table 5 lying mostly around 20–30% with a maximum shrinkage of 49%. Overall, even though the model requires the estimation of a relatively large number of parameters, identifiability seems to be no issue. The sensitivity analysis of this model led to a maximal distance of 0.49 to the reference ANC trajectory even in the most extreme case with the majority of the deviations of the disturbed trajectories lying well below that. This indicates that fixing these parameters does not influence the resulting ANC trajectories considerably, with the absorption rate ${\mathrm{k}}_{\mathrm{a}}^{6\mathrm{MP}}$ being the most influential parameter of the fixed ones. Therefore, the model would benefit from a better data basis with the ability to determine the absorption rate with a smaller confidence interval.

**Figure 4:**
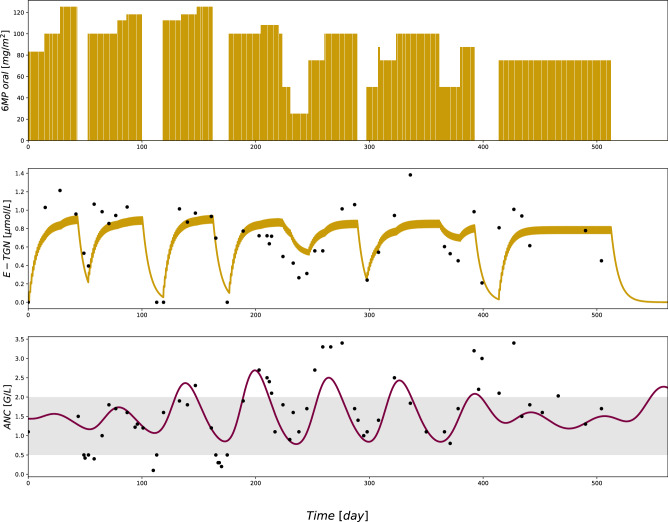
Treatment schedule, E-TGN and ANC trajectories resulting from the final PKPD model for one patient showing the relation between 6MP doses, E-TGN concentrations and the ANC.

Compared to the results of Jost et al.^10^, the ANC trajectories resulting from our model display the same oscillations (cf. Figure 4 with the trajectories of one patient and Supplementary Figures S23 online). In contrast to the model of Jost et al.^10^ including E-TGN observations and therefore individual parameters for the intracellular pharmacokinetics, we see the RMSEs and MAEs of the individual ANC predictions resulting from the cross-validation varying less than when relying on values from the literature for the 6MP PK submodel as in Jost et al.^10^ indicating that individualizing this part of the model leads to more robust predictions even with fewer observations of E-TGN and ANC per patient. A similar advantage of the newly developed PKPD model holds true when comparing the results of the population predictions with the ANC observations of the individual patients: the model $PKP{D}_{lin,mm}^{6MP}$ leads to distinctly lower RMSEs and MAEs, suggesting a better fit of the model predictions to the patient observations if no prior individual information is available. Overall, our model testifies to the quality (i.e., description of the so-called ground truth) of the model of Jost et al. when sufficient data is available, but performs better in circumstances with limited data availability^10^. This advantage has to be weighed against possible disadvantages of model complexification, such as estimates becoming more uncertain when increasing a model’s effective dimension^38^.

While our model seems to replicate the dynamics of ALL maintenance therapy well overall, its ability to reach the minima and maxima of the ANC is limited, in contrast to e.g., models of acute myeloid leukemia (AML) predicting especially the nadir of treatment cycles accurately^39, 40^. The reason for this might be differences in population with the AML models using data of adult patients versus data of still developing pediatric patients, and time horizons with the treatment cycles of AML lasting around 30–50 days versus 1–2 years of maintenance therapy. With the Jost et al. model displaying the same characteristics^10^, this might point to general limits of this modeling approach for ALL maintenance therapy. This property of the model is especially relevant when simulating new treatment schedules or when using the model for optimization. The clinical applicability of PKPD models also depend on the models’ ability to predict the timing of samples and the expected effect on ANC. It is important to note that the trajectories of the ANC are not only influenced by the linear effect function but also by the nonlinear feedback mechanism, which necessitates more involved numerical studies to explore this relationship. We plan to address this in a subsequent paper.

One possible extension of the here developed PKPD model could be inspired by the results of Korell et al.^41^ In their study, they compare the intracellular pharmacokinetics of MTX in the red blood cells and other cell lines and come to the conclusion that the dynamics of MTX in the red blood cells differ significantly from those in the white blood cells. While our final PKPD model does not include a MTX PK submodel, the same might hold for 6MP and would require further research into the intracellular pharmacokinetics of TGN in the white blood cells to integrate this into the PKPD model. DNA-TG in leukocytes is associated with E-TGN, E-MTX and E-MMP^42^. Furthermore, DNA-TG has been associated with the risk of relapse in patients with positive measurable residual disease at the end of induction therapy^42, 43^. However, the impact of DNA-TG on ANC has not yet been reliably modelled, and an optimal level of DNA-TG remains to be determined before DNA-TG can be used as a target for TDM^3, 44^.

## Conclusion

We developed a full pharmacokinetic–pharmacodynamic model that relies on observations of E-TGN and ANC to predict the effect maintenance therapy has on the patient-specific dynamics of the ANC. The results of the parameters estimation of this model are mostly comparable to those of Jost et al.^10^ with a structurally similar model replicating the same dynamics and indicating that at this point, this model structure is the most promising for ALL maintenance therapy. The newly developed model shows a better fit to the patient-wise observations in situations where less data is available as is often the case in clinical practice. This model now allows comprehensive analysis of the effects resulting from the modeled dynamics to generate hypotheses for the optimization of adapted treatment regimens through systematic simulation studies.

## Supplementary Information

Supplementary Information 1.

Supplementary Information 2.

## Data Availability

The estimated patient parameters of this study are included in the Supplementary Information. The original data analysed in this study is not publicly available to preserve patient confidentiality.

## Code availability

All relevant Python and NONMEM code for the model analysis and simulation are available from the authors upon reasonable request.
