# Supplementary material for: Pharmacokinetic–pharmacodynamic modeling of maintenance therapy for childhood acute lymphoblastic leukemia
Source: Sci Rep. 2023 Jul 20;13:11749. doi: 10.1038/s41598-023-38414-0 (PMC10359452; doi:10.1038/s41598-023-38414-0)
Supplement: Supplementary file 1 — Supplementary Information 1. [file 41598_2023_38414_MOESM1_ESM.pdf]

Pharmacokinetic-Pharmacodynamic Modeling of Maintenance Therapy for Childhood Acute Lymphoblastic Leukemia

Anna Gebhard[1], Patrick Lilienthal[1], Markus Metzler[2], Manfred Rauh[2], Sebastian Sager[1], Kjeld Schmiegelow[3,4], Linea Natalie Toksvang[3*], Jakob Zierk[2*]

[1]MathOpt group, Institute of Mathematical Optimization, Faculty of Mathematics, Otto von Guericke University Magdeburg, Magdeburg, Germany

[2]Department of Pediatrics and Adolescent Medicine, University Hospital Erlangen, Erlangen, Germany

[3]Department of Pediatrics and Adolescent Medicine, University Hospital Rigshospitalet, Copenhagen, Denmark

[4]Institute of Clinical Medicine, Faculty of Medicine, University of Copenhagen, Copenhagen, Denmark

*These authors contributed equally

Supplementary Information

A Visualization and derivation of model structures

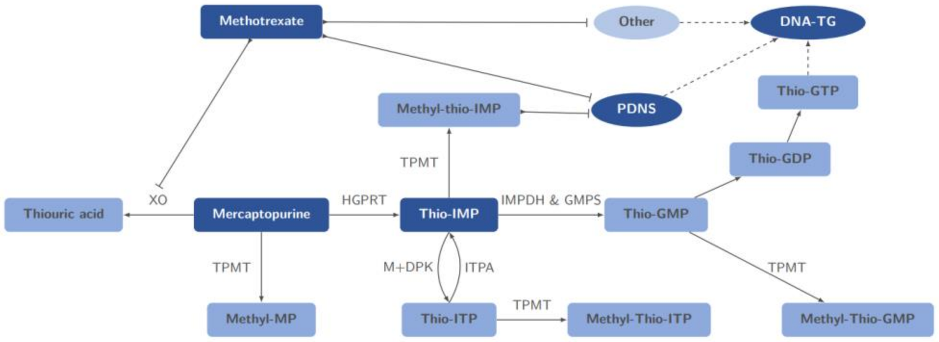

Supplementary Figure S1: Simplified version of Figure 3 in Schmiegelow et al. visualizing the MTX and 6MP metabolism and interaction. [19]

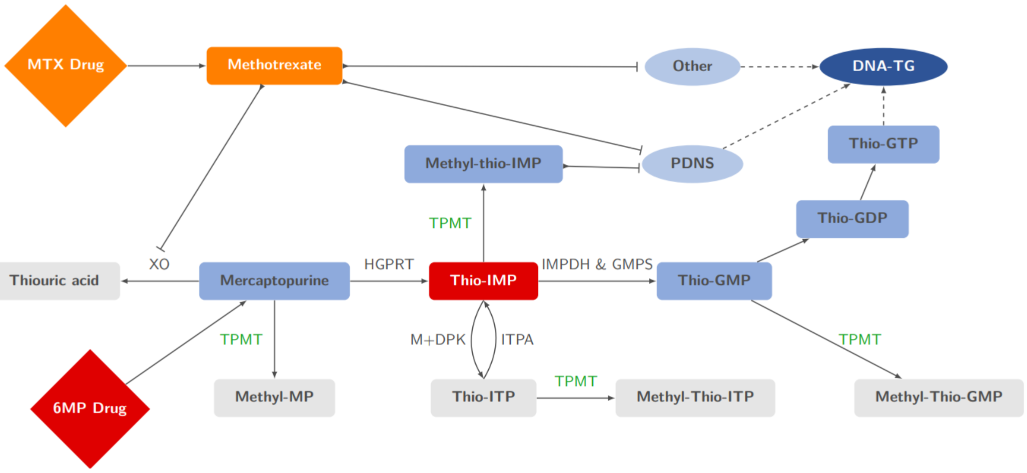

Supplementary Figure S2: MTX and 6MP metabolism and interaction with the relevant paths for the model development process highlighted.

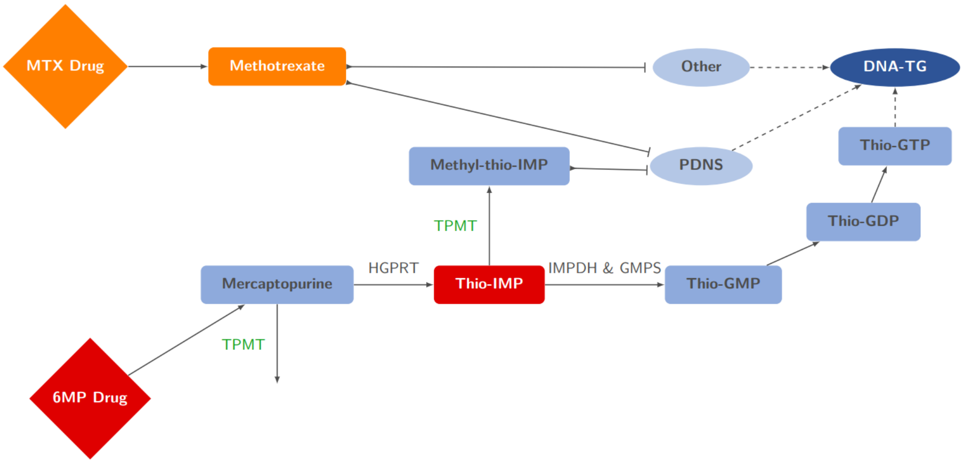

Supplementary Figure S3: MTX and 6MP metabolism and interaction with the less relevant paths for the model development process omitted.

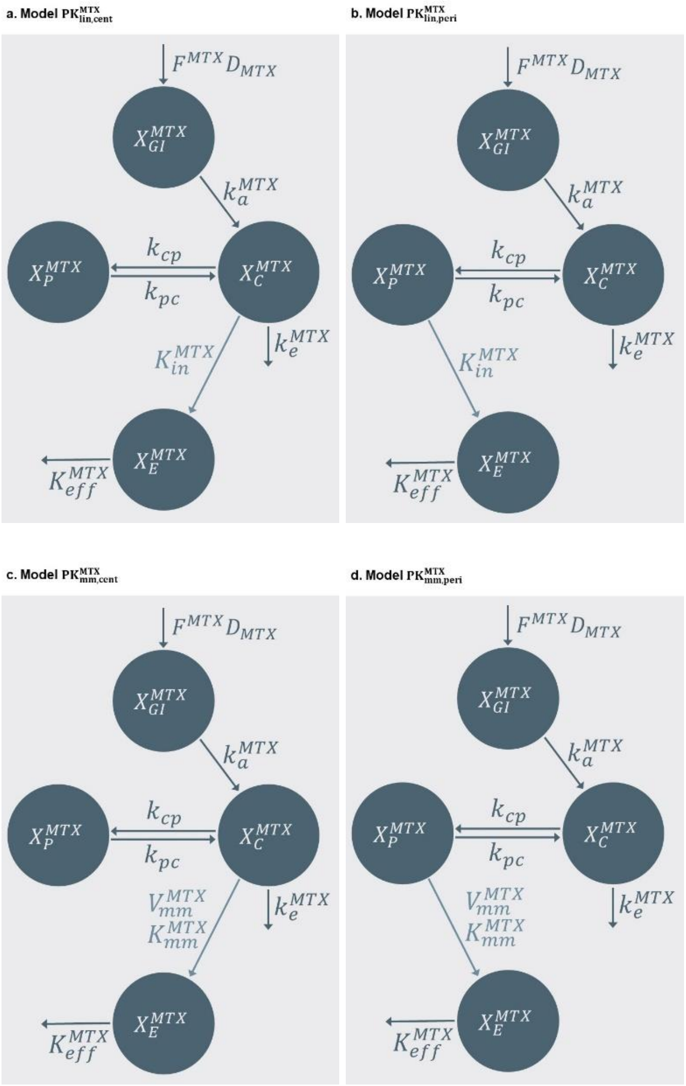

Supplementary Figure S4: The four structurally different MTX PK models, a) and b) with linear kinetics for the influx of MTX in the red blood cells, c) and d) with Michaelis-Menten kinetics. The first column shows the models with the influx based on the central compartment and the second column based on the peripheral compartment. (See equations MTX1-4.)

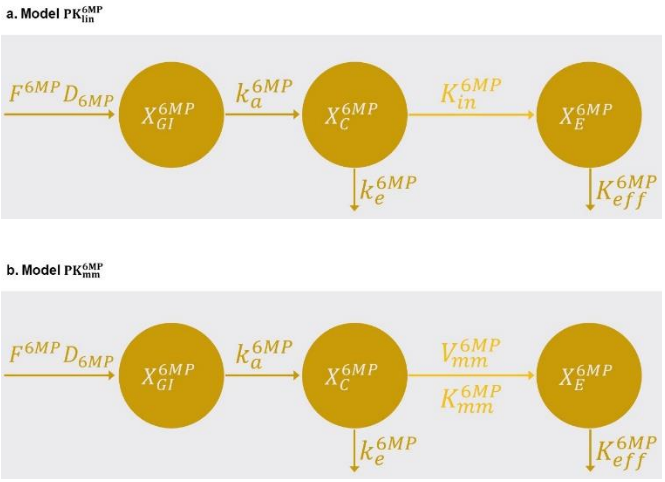

Supplementary Figure S5: The two structurally different6MP PK models, a) with linear kinetics for the influx of 6MP in the red blood cells, b) with Michaelis-Menten kinetics. (See equations 6MP1-3.)

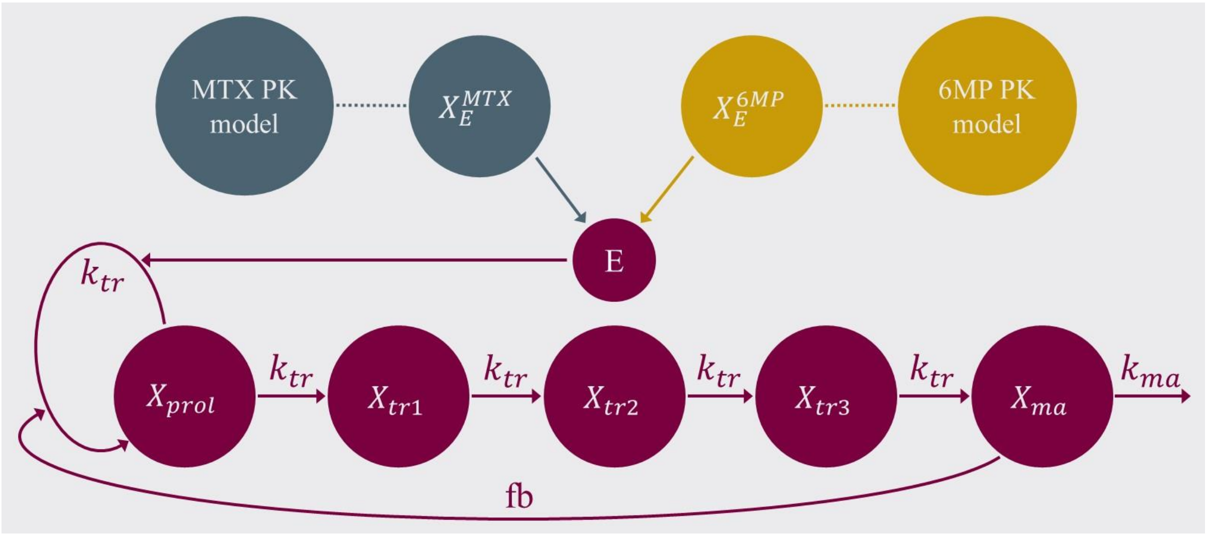

Supplementary Figure S6: The PKPD model variants with the PK model variants as previously described and the Friberg et al. model as the PD model. [15] (See equations PKPD1-7.)

B Overview and calculation of fixed PK parameters for the MTX PK model

Supplementary Table S1: Parameters of the plasma pharmacokinetics of the MTX PK models. BSA is calculated according to the Mosteller formula. [45]

Model	Parameter	Value	Source
PK_{fix}^{MTX}	Bioavailability F^{MTX}	0.45	[9]
	Absorption rate k_a^{MTX}	26.64 1/day	[9]
	Intercompartmental rate k_{cp}	0.9888 1/day	[21]
	Intercompartmental rate k_{pc}	2.64 1/day	[21]
	Elimination rate k_e^{MTX}	14.2944 1/day	[21]
	Central volume V_C^{MTX}	11.606 L/m^2	[21]
	Peripheral volume V_P^{MTX}	4.347 L/m^2	[21]
PK_{bio}^{MTX}	Bioavailability F^{MTX}	$1 - \frac{0.77 \cdot MTX}{15.01+MTX}$ (MTX in mg/m^2)	[23]
PK_{ind}^{MTX}	Bioavailability F^{MTX}	0.45	[9]
	Absorption rate k_a^{MTX}	26.64 1/day	[9]

	Intercompartmental rate k_{cp}	$\frac{9.84}{0.36 \cdot WEIGHT}$ 1/day (WEIGHT in kg)	[24]
	Intercompartmental rate k_{pc}	3.075 1/day	[24]
	Elimination rate k_e^{MTX}	$\frac{156 \cdot BSA^{0.62}}{0.36 \cdot WEIGHT}$ 1/day (BSA in m^2, WEIGHT in kg)	[24]
	Central volume V_C^{MTX}	$\frac{0.36 \cdot WEIGHT}{BSA}$ L/m^2 (BSA in m^2, WEIGHT in kg)	[24]
	Peripheral volume V_P^{MTX}	$\frac{3.2}{BSA}$ L/m^2 (BSA in m^2, WEIGHT in kg)	[24]

Supplementary Table S1 shows the values of the fixed parameters in the MTX PK models, which were calculated in the following way:

For the models PK_{fix}^{MTX}, the intercompartmental rates k_{cp} and k_{pc}, the elimination rate k_e^{MTX} and the central volume V_C^{MTX} are the mean of the corresponding five values listed in Table 2 in Panetta et al. converted to 1/day. [21] The elimination rate k_e^{MTX} is the mean of CL/V of the corresponding five values listed in Table 2 in Panetta et al. converted to 1/day. [21] The peripheral volume V_P^{MTX} is calculated as $V_P^{MTX} = \frac{V_C^{MTX} \cdot k_{12}}{k_{21}}$.

For the models PK_{ind}^{MTX}, the intercompartmental rates k_{cp} and k_{pc} are $Q/(V_c \cdot WEIGHT)$ and Q/V_p listed in Table 2 in Ogungbenro et al., respectively, and converted to 1/day. [24] The elimination rate k_e^{MTX} is $CL/(V_c \cdot WEIGHT)$ taken from Table 2 in Ogungbenro et al. converted to 1/day. [24] The central volume V_C^{MTX} and the peripheral volume V_P^{MTX} are the corresponding volumes in Table 2 in Ogungbenro et al. converted to L/m^2 by using the covariates WEIGHT and BSA for scaling. [24]

Parameters not listed here were taken directly from the literature source and not converted in any way.

C Overview and calculation of fixed PK parameters for the 6MP PK model

Supplementary Table S2: Parameters of the plasma pharmacokinetics of the 6MP PK models.

Model	Parameter	Value	Source
PK^{6MP}	Bioavailability F^{6MP}	0.12	[46]
	Absorption rate k_a^{6MP}	21.07 1/day	[30]
	Elimination rate k_e^{6MP}	15.4 1/day	[30]
	Central volume V_C^{6MP}	20.1911 L/m^2	[30]

Supplementary Table S2 shows the values of the fixed parameters in the 6MP PK models, which were calculated in the following way:

The bioavailability F^{6MP} is taken directly from Brunton et al. [46]

The absorption rate k_a^{6MP}, the elimination rate k_e^{6MP} and the central volume V_C^{6MP} are calculated by using the means of in Table 1 in Lennard et al., [30] the stated dose of 75 mg/m^2 and the explicit solution of the ODE system of a 1-CTM-model:

$$C(t) = \frac{F^{6MP} \cdot Dose_{6MP}}{V_C^{6MP}} \cdot k_a^{6MP} \cdot \frac{\exp(-k_e^{6MP} \cdot t) - \exp(-k_a^{6MP} \cdot t)}{k_a^{6MP} - k_e^{6MP}}$$

Which implies the following relationships:

$$k_e^{6MP} = \frac{\ln(2)}{t_{1/2}}$$

$$t_{max} = \frac{\ln(k_a^{6MP}) - \ln(k_e^{6MP})}{k_a^{6MP} - k_e^{6MP}}$$

$$V_C^{6MP} = \frac{F^{6MP} \cdot D}{C_{max}} \cdot k_a^{6MP} \cdot \frac{\exp(-k_e^{6MP} \cdot t_{\max}) - \exp(-k_a^{6MP} \cdot t_{max})}{k_a^{6MP} - k_e^{6MP}}$$

The absorption rate k_a^{6MP} and the elimination rate k_e^{6MP} were converted to 1/day, and the central volume V_C^{6MP} to L/m^2.

D Overview of model variants

Supplementary Table S3: Overview of all model variants including model names, a short description of the model and the equations used in the corresponding ODE system.

Model name	Model description	Equations used
**MTX PK model**		
$PK_{fix,mm,cent}^{MTX}$	Influx into the red blood cells from the central compartment using Michaelis-Menten kinetics, fixed parameters as in PK_{fix}^{MTX} in Supplementary Table S1 online.	MTX1-3, MTX4b
$PK_{fix,bio,mm,cent}^{MTX}$	Influx into the red blood cells from the central compartment using Michaelis-Menten kinetics, fixed parameters as in PK_{fix}^{MTX} in Supplementary Table S1 online, but with dose-dependent bioavailability as in PK_{bio}^{MTX}.	MTX1-3, MTX4b
$PK_{fix,mm,peri}^{MTX}$	Influx into the red blood cells from the peripheral compartment using Michaelis-Menten kinetics, fixed parameters as in PK_{fix}^{MTX} in Supplementary Table S1 online.	MTX1-3, MTX4d
$PK_{fix,bio,mm,peri}^{MTX}$	Influx into the red blood cells from the peripheral compartment using Michaelis-Menten kinetics, fixed parameters as in PK_{fix}^{MTX} in Supplementary Table S1 online, but with dose-dependent bioavailability as in PK_{bio}^{MTX}.	MTX1-3, MTX4d
$PK_{ind,mm,cent}^{MTX}$	Influx into the red blood cells from the central compartment using Michaelis-Menten kinetics, fixed parameters as in PK_{ind}^{MTX} in TSupplementary Table S1 online.	MTX1-3, MTX4b
$PK_{ind,bio,mm,cent}^{MTX}$	Influx into the red blood cells from the central compartment using Michaelis-Menten kinetics, fixed parameters as in PK_{ind}^{MTX} in Supplementary Table S1 online, but with dose-dependent bioavailability as in PK_{bio}^{MTX}.	MTX1-3, MTX4b
$PK_{ind,mm,peri}^{MTX}$	Influx into the red blood cells from the peripheral compartment using Michaelis-Menten kinetics, fixed parameters as in PK_{ind}^{MTX} in Supplementary Table S1 online.	MTX1-3, MTX4d
$PK_{ind,bio,mm,peri}^{MTX}$	Influx into the red blood cells from the peripheral compartment using Michaelis-Menten kinetics, fixed parameters as in PK_{ind}^{MTX} in Supplementary Table S1 online, but with dose-dependent bioavailability as in PK_{bio}^{MTX}.	MTX1-3, MTX4d
$PK_{fix,lin,cent}^{MTX}$	Influx into the red blood cells from the central compartment using linear kinetics, fixed parameters as in PK_{fix}^{MTX} in Supplementary Table S1 online.	MTX1-3, MTX4a
$PK_{fix,bio,lin,cent}^{MTX}$	Influx into the red blood cells from the central compartment using linear kinetics, fixed parameters as in PK_{fix}^{MTX} in Supplementary Table S1 online, but with dose-dependent bioavailability as in PK_{bio}^{MTX}.	MTX1-3, MTX4a
$PK_{fix,lin,peri}^{MTX}$	Influx into the red blood cells from the peripheral compartment using linear kinetics, fixed parameters as in PK_{fix}^{MTX} in Supplementary Table S1 online.	MTX1-3, MTX4c
$PK_{fix,bio,lin,peri}^{MTX}$	Influx into the red blood cells from the peripheral compartment using linear kinetics, fixed parameters as in PK_{fix}^{MTX} in Supplementary Table S1 online, but with dose-dependent bioavailability as in PK_{bio}^{MTX}.	MTX1-3, MTX4c
$PK_{ind,lin,cent}^{MTX}$	Influx into the red blood cells from the central compartment using linear kinetics, fixed parameters as in PK_{ind}^{MTX} in Supplementary Table S1 online.	MTX1-3, MTX4a
$PK_{ind,bio,lin,cent}^{MTX}$	Influx into the red blood cells from the central compartment using linear kinetics, fixed parameters as in PK_{ind}^{MTX} in Supplementary Table S1 online, but with dose-dependent bioavailability as in PK_{bio}^{MTX}.	MTX1-3, MTX4a
$PK_{ind,lin,peri}^{MTX}$	Influx into the red blood cells from the peripheral compartment	MTX1-3, MTX4c

	using linear kinetics, fixed parameters as in PK_{ind}^{MTX} in Supplementary Table S1 online.	
$PK_{ind,bio,lin,peri}^{MTX}$	Influx into the red blood cells from the peripheral compartment using linear kinetics, fixed parameters as in PK_{ind}^{MTX} in Supplementary Table S1 online, but with dose-dependent bioavailability as in PK_{bio}^{MTX}.	MTX1-3, MTX4c
$PK_{fix,mm,cent,pop}^{MTX}$	Influx into the red blood cells from the central compartment using Michaelis-Menten kinetics, fixed parameters as in PK_{fix}^{MTX} in Supplementary Table S1 online. Parameter K_{mm}^{MTX} without interindividual variability.	MTX1-3, MTX4b
$PK_{fix,bio,lin,peri,ae}^{MTX}$	Influx into the red blood cells from the peripheral compartment using linear kinetics, fixed parameters as in PK_{fix}^{MTX} in Supplementary Table S1 online, but with dose-dependent bioavailability as in PK_{bio}^{MTX}. Purely additive residual error model.	MTX1-3, MTX4c
$PK_{fix,bio,lin,peri,pe}^{MTX}$	Influx into the red blood cells from the peripheral compartment using linear kinetics, fixed parameters as in PK_{fix}^{MTX} in Supplementary Table S1 online, but with dose-dependent bioavailability as in PK_{bio}^{MTX}. Purely proportional residual error model.	MTX1-3, MTX4c
$PK_{fix,bio,lin,peri,eps}^{MTX}$	Influx into the red blood cells from the peripheral compartment using linear kinetics, fixed parameters as in PK_{fix}^{MTX} in Supplementary Table S1 online, but with dose-dependent bioavailability as in PK_{bio}^{MTX}. Initial values with the possibility of varying similarly to the residual error model: $X_E^{MTX}(0) = (INIMTX - \sigma_2^{MTX} \cdot \eta_2^{INIMTX})/(1 + \sigma_1^{MTX} \cdot \eta_1^{INIMTX})$.	MTX1-3, MTX4c
**6MP PK model**		
PK_{mm}^{6MP}	Influx into the red blood cells using Michaelis-Menten kinetics, fixed parameters as in PK^{6MP} in Supplementary Table S2 online.	6MP1-2, 6MP3b
PK_{lin}^{6MP}	Influx into the red blood cells using Michaelis-Menten kinetics, fixed parameters as inPK^{6MP} in Supplementary Table S2 online.	6MP1-2, 6MP3a
$PK_{mm,pop}^{6MP}$	Influx into the red blood cells using Michaelis-Menten kinetics, fixed parameters as in PK^{6MP} in Supplementary Table S2 online. Parameter K_{mm}^{6MP} without interindividual variability.	6MP1-2, 6MP3b
$PK_{mm,pop,ae}^{6MP}$	Influx into the red blood cells using Michaelis-Menten kinetics, fixed parameters as in PK^{6MP} in Supplementary Table S2 online. Purely additive residual error model.	6MP1-2, 6MP3b
$PK_{mm,pop,pe}^{6MP}$	Influx into the red blood cells using Michaelis-Menten kinetics, fixed parameters as in PK^{6MP} in Supplementary Table S2 online. Purely proportional residual error model.	6MP1-2, 6MP3b
$PK_{mm,pop,eps}^{6MP}$	Influx into the red blood cells using Michaelis-Menten kinetics, fixed parameters as in PK^{6MP} in Supplementary Table S2 online. Initial values with the possibility of varying similarly to the residual error model: $X_E^{6MP}(0) = (INITGN - \sigma_2^{6MP} \cdot \eta_2^{INITGN})/(1 + \sigma_1^{6MP} \cdot \eta_1^{INITGN})$.	6MP1-2, 6MP3b
**PKPD model**		
$PKPD^{Jost}$	The model described in Jost et al. with the initial values as described in section 2.2.3. [10]	Equations 1-3 in [10]
$PKPD_{lin}^{6MP}$	PK models $PK_{fix,bio,lin,peri}^{MTX}$ and PK_{lin}^{6MP} combined with an effect function based on E-TGN.	MTX1-3, MTX4c, 6MP1-2, 6MP3a, PKPD1-6, PKPD7a
$PKPD_{lin,mm}^{6MP}$	PK models $PK_{fix,bio,lin,peri}^{MTX}$ and $PK_{mm,pop}^{6MP}$ combined with an effect function based on E-TGN.	MTX1-3, MTX4c, PKPD1-6, PKPD7a
$PKPD_{lin}^{6MP,MTX}$	PK models $PK_{fix,bio,lin,peri}^{MTX}$ and PK_{lin}^{6MP} combined with an effect function based on a combination of E-MTX and E-TGN.	MTX1-3, MTX4c, 6MP1-2, 6MP3a, PKPD1-6, PKPD7b
$PKPD_{lin,mm}^{6MP,MTX}$	PK models $PK_{fix,bio,lin,peri}^{MTX}$ and $PK_{mm,pop}^{6MP}$ combined with an effect function based on a combination of E-MTX and E-TGN.	MTX1-3, MTX4c, 6MP1-2, 6MP3b, PKPD1-6, PKPD7b

E Detailed Results for the MTX PK model

Supplementary Table S4: Objective function values and RMSEs and MAEs of the different MTX PK models. RMSEs and MAEs of the patient trajectories computed with the individual patient parameters are calculated patientwise, the reported values are the means and medians, standard deviation in brackets.

	$PK_{fix,mm,cent}^{MTX}$	$PK_{fix,bio,mm,cent}^{MTX}$	$PK_{fix,mm,peri}^{MTX}$	$PK_{fix,bio,mm,peri}^{MTX}$
**OBJ**	-37,421	-37,416	-37,422	-37,421
**Errors of the individual predictions**				
Median of RMSEs	0.0043 (0.0028)	0.0042 (0.0028)	0.0042 (0.0028)	0.0042 (0.0028)
Mean of RMSEs	0.0048 (0.0028)	0.0048 (0.0028)	0.0048 (0.0028)	0.0048 (0.0028)
Median of MAEs	0.0033 (0.0022)	0.0033 (0.0022)	0.0033 (0.0022)	0.0033 (0.0022)
Mean of MAEs	0.0037 (0.0022)	0.0037 (0.0022)	0.0036 (0.0022)	0.0037 (0.0022)
	$PK_{ind,mm,cent}^{MTX}$	$PK_{ind,bio,mm,cent}^{MTX}$	$PK_{ind,mm,peri}^{MTX}$	$PK_{ind,bio,mm,peri}^{MTX}$
**OBJ**	-37,393	-37,360	-37,385	-37,397
**Errors of the individual predictions**				
Median of RMSEs	0.0043 (0.0028)	0.0043 (0.0028)	0.0043 (0.0028)	0.0042 (0.0028)
Mean of RMSEs	0.0048 (0.0028)	0.0048 (0.0028)	0.0048 (0.0028)	0.0048 (0.0028)
Median of MAEs	0.0033 (0.0022)	0.0033 (0.0022)	0.0033 (0.0022)	0.0033 (0.0022)
Mean of MAEs	0.0037 (0.0022)	0.0037 (0.0022)	0.0037 (0.0022)	0.0037 (0.0022)
	$PK_{fix,lin,cent}^{MTX}$	$PK_{fix,bio,lin,cent}^{MTX}$	$PK_{fix,lin,peri}^{MTX}$	$PK_{fix,bio,lin,peri}^{MTX}$
**OBJ**	-37,181	-37,354	-37,184	-37,359
**Errors of the individual predictions**				
Median of RMSEs	0.0043 (0.0028)	0.0042 (0.0028)	0.0043 (0.0028)	0.0042 (0.0028)
Mean of RMSEs	0.0048 (0.0028)	0.0047 (0.0028)	0.0048 (0.0028)	0.0047 (0.0028)
Median of MAEs	0.0033 (0.0022)	0.0033 (0.0022)	0.0033 (0.0022)	0.0033 (0.0022)
Mean of MAEs	0.0037 (0.0022)	0.0036 (0.0022)	0.0037 (0.0022)	0.0036 (0.0022)
	$PK_{ind,lin,cent}^{MTX}$	$PK_{ind,bio,lin,cent}^{MTX}$	$PK_{ind,lin,peri}^{MTX}$	$PK_{ind,bio,lin,peri}^{MTX}$
**OBJ**	-37,102	-37,276	-37,105	-37,280
**Errors of the individual predictions**				
Median of RMSEs	0.0043 (0.0028)	0.0042 (0.0028)	0.0043 (0.0028)	0.0042 (0.0028)
Mean of RMSEs	0.0048 (0.0028)	0.0047 (0.0028)	0.0047 (0.0028)	0.0047 (0.0028)
Median of MAEs	0.0033 (0.0022)	0.0033 (0.0022)	0.0033 (0.0022)	0.0033 (0.0022)
Mean of MAEs	0.0036 (0.0022)	0.0036 (0.0022)	0.0036 (0.0022)	0.0036 (0.0022)
	$PK_{fix,mm,cent,pop}^{MTX}$	$PK_{fix,bio,lin,peri,ae}^{MTX}$	$PK_{fix,bio,lin,peri,pe}^{MTX}$	$PK_{fix,bio,lin,peri,eps}^{MTX}$
**OBJ**	-37,421	-37,124	-36,963	-36,463
**Errors of the individual predictions**				
Median of RMSEs	0.0043 (0.0028)	0.0043 (0.0027)	0.0043 (0.0029)	0.0045 (0.0027)
Mean of RMSEs	0.0048 (0.0028)	0.0048 (0.0027)	0.0048 (0.0029)	0.0050 (0.0027)
Median of MAEs	0.0033 (0.0022)	0.0033 (0.0021)	0.0033 (0.0022)	0.0037 (0.0021)
Mean of MAEs	0.0037 (0.0022)	0.0036 (0.0021)	0.0037 (0.0022)	0.0041 (0.0021)

The computed errors in Supplementary Table S4 online show almost no differences between the models, but the corresponding objective function values do. For the model structure with Michaelis-Menten kinetics for the influx of MTX into the red blood cells listed in the first part, we find the lowest objective function values for $PK_{fix,mm,cent}^{MTX}$, $PK_{fix,mm,peri}^{MTX}$ and $PK_{fix,bio,mm,peri}^{MTX}$. As the model extensions with adjusted bioavailability and

basing the influx on the peripheral compartment did not lead to better results than the base variant with fixed bioavailability and the influx based in the central compartment, $PK_{fix,mm,cent}^{MTX}$ would be preferred if only looking at this structural model. The second part in Supplementary Table S4 online compares the model variants with linear influx kinetics, leading to $PK_{fix,bio,lin,peri}^{MTX}$ as the model with the lowest objective function, but with a value of -37,359, it is still higher than the one of model $PK_{fix,mm,cent}^{MTX}$ with a value of -37,421.

While model $PK_{fix,mm,cent}^{MTX}$ has the lowest objective function value, having a closer look at the results of the parameter estimation revealed that the 95%-confidence interval (CI) for the coefficient of variation of K_{mm}^{MTX} of this model covers 0. We therefore estimated the model again with the interindividual variability for K_{mm}^{MTX} removed, resulting in one common K_{mm}^{MTX} parameter for all individual patients. As the third part in Supplementary Table S4 online shows, model $PK_{fix,mm,cent,pop}^{MTX}$ led to the same objective function value, RMSEs and MAEs of the individual predictions as model $PK_{fix,mm,cent}^{MTX}$, without any 95%-CI of the interindividual variabilities covering 0.

We then compared the results of the cross-validation for both of the structurally different model variants $PK_{fix,mm,cent,pop}^{MTX}$ and $PK_{fix,bio,lin,peri}^{MTX}$. Supplementary Table S5 online compares the results for the parameter estimation of $PK_{fix,mm,cent,pop}^{MTX}$ for all data and for the cross-validation set, showing only slight differences in the RMSEs and MAEs of the individual predictions, but significant changes in the resulting parameters. Most evidently, K_{mm}^{MTX} is almost two orders of magnitude smaller when estimating with the cross-validation data set, with its relative standard error increasing to 84%. Supplementary Figure S7 online depicts the changes in the fixed effect parameters V_{mm}^{MTX} and K_{eff}^{MTX} by comparing all individual patient parameters resulting from estimating using the whole data set and using the cross-validation data set, showing huge differences between both.

Next, we tested the final model $PK_{fix,bio,lin,peri}^{MTX}$ against four further variations: we replaced the additive-proportional residual error model with a purely additive ($PK_{fix,bio,lin,peri,ae}^{MTX}$) and a purely proportional one ($PK_{fix,bio,lin,peri,pe}^{MTX}$), and we swapped the initialization of the E-MTX compartment by an observation with either a direct estimation of the value, or the possibility of varying similarly to the residual error model ($PK_{fix,bio,lin,peri,eps}^{MTX}$). The model variant with the estimated initial value led to a result with implausible parameter values and NONMEM not being able to estimate the covariance matrix. It is therefore not included here. All three other models resulted in a higher objective function value than $PK_{fix,bio,lin,peri}^{MTX}$, cf. Supplementary Table S4 online.

Supplementary Table S5: Parameter values of the model $PK_{fix,mm,cent,pop}^{MTX}$ estimated with the whole and the cross-validation data set, relative standard error (RSE) of fixed effect parameters and residual unexplained variability in brackets. RMSEs and MAEs of the patient trajectories computed with the individual patient parameters are each calculated for the whole data set with the standard deviation in brackets. Coefficients of variation (CV) are calculated as $\sqrt{exp(\omega^2) - 1}$ with ω^2 as the variance of the interindividual variability estimated by NONMEM, and the 95%-CI being calculated accordingly.

	$PK_{fix,mm,cent,pop}^{MTX}$	$PK_{fix,mm,cent,pop}^{MTX}$, **cross-validation**
**Fixed effects parameters with RSE in %**		
V_{mm}^{MTX} [µmol/L/day]	0.015 (7)	0.0032 (23)
K_{mm}^{MTX} [µmol/L]	0.075 (13)	0.00085 (84)
K_{eff}^{MTX} [1/day]	0.020 (4)	0.023 (6)
**CV with 95%-CI, η-Shrinkage in % and p-values**		
V_{mm}^{MTX}	29 [25, 33], 31, 0.28	30 [26, 35], 33, 0.20
K_{eff}^{MTX}	27 [23, 31], 33, 0.59	18 [11, 23], 55, 0.47
**Residual unexplained variability**		
Additive residual error	0.000019 (8)	0.000021 (9)
Proportional residual error	0.023 (7)	0.0088 (24)
**Errors of the individual predictions**		
Median of RMSEs	0.0043 (0.0028)	0.0053 (0.0044)
Mean of RMSEs	0.0048 (0.0028)	0.0063 (0.0044)
Median of MAEs	0.0033 (0.0022)	0.0039 (0.0034)
Mean of MAEs	0.0037 (0.0022)	0.0048 (0.0034)

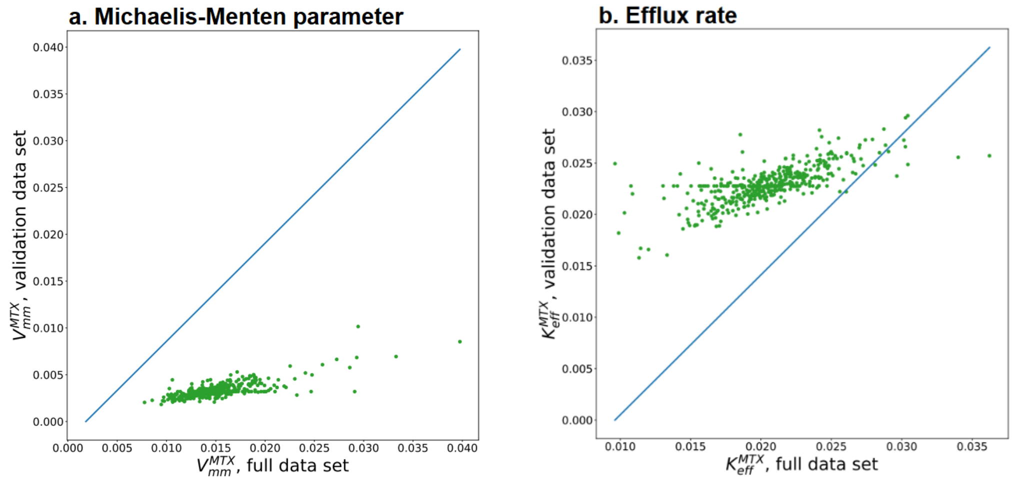

Supplementary Figure S7: Scatterplots of the individual patient parameters V_{mm}^{MTX} *and* K_{eff}^{MTX} *for the model* $PK_{fix,mm,cent,pop}^{MTX}$*, parameters estimated with the full data set on the horizontal axis and with the validation data set on the vertical axis.*

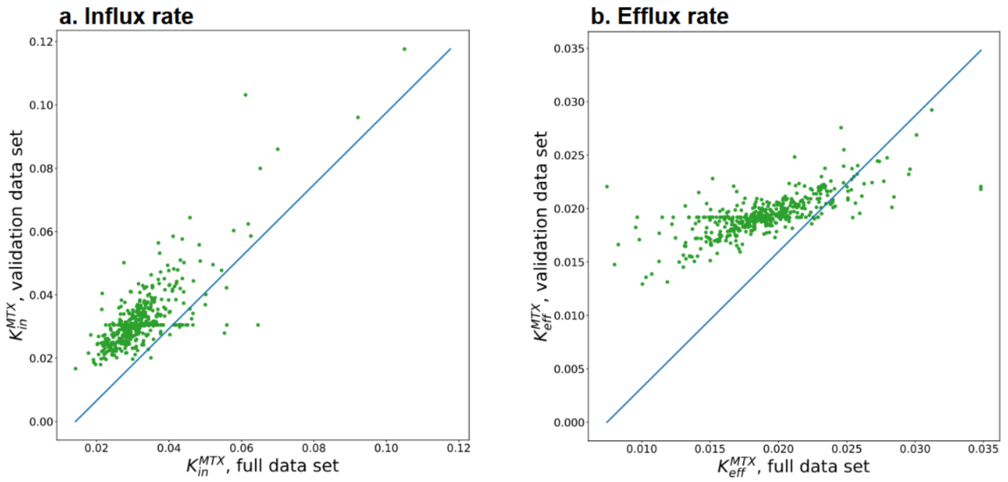

Supplementary Figure S8: Scatterplots of the individual patient parameters K_{in}^{MTX} *and* K_{eff}^{MTX} *for the* $PK_{fix,bio,lin,peri}^{MTX}$ *model, parameters estimated with the full data set on the horizontal axis and with the validation data set on the vertical axis.*

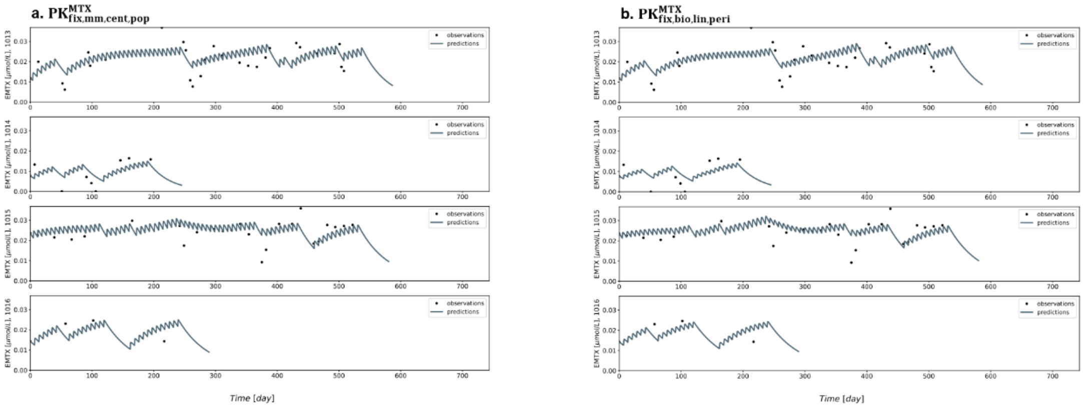

Supplementary Figure S9: Individual trajectories of four patients.

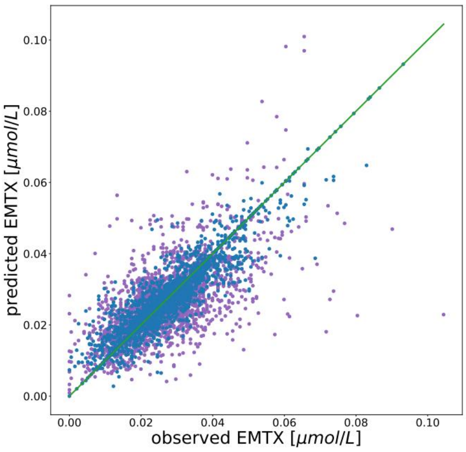

Supplementary Figure S10: Goodness-of-fit plot of observed and by the model $PK^{MTX}_{fix,bio,lin,peri}$ predicted E-MTX values. Estimation with the cross-validation data set, where blue are in-sample and purple out-of-sample observations.

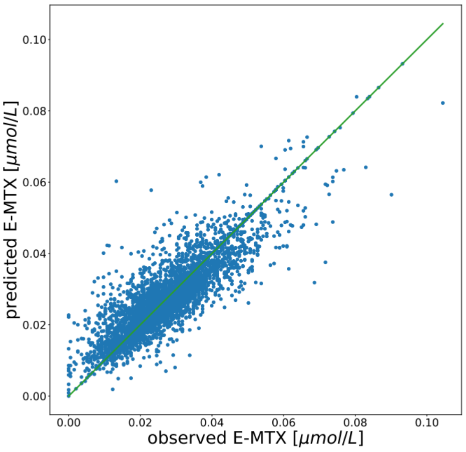

Supplementary Figure S11: Goodness-of-fit plot of observed and by the model $PK^{MTX}_{fix,bio,lin,peri}$ predicted E-MTX values, estimation using the whole data set.

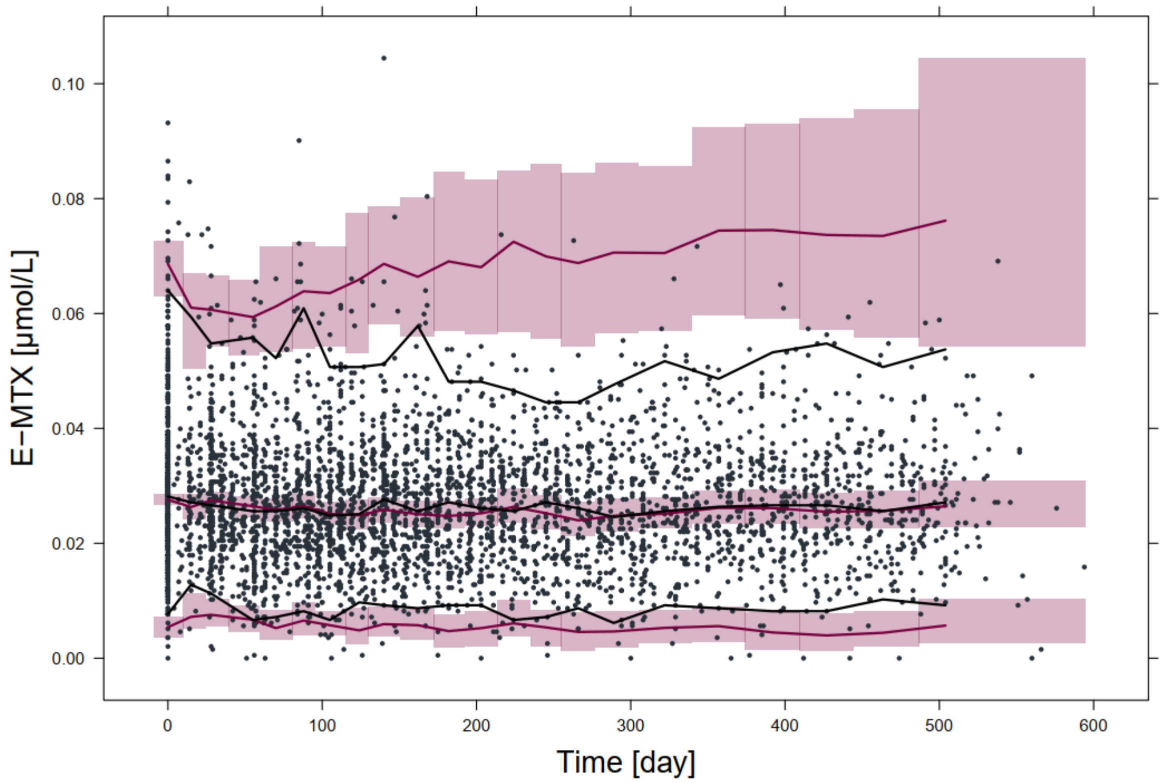

Supplementary Figure S12: Visual predictive check with 1000 simulations of the model $PK_{fix,bio,lin,peri}^{MTX}$. The grey dots represent the observed E-MTX values, the black lines the corresponding median, 2.5th and 97.5th percentiles. The red lines show the median, 2.5th and 97.5th percentiles of the model predictions, the shaded areas represent the corresponding 95% confidence intervals.

F Detailed Results for the 6MP PK model

Supplementary Table S6: Objective function values and RMSEs and MAEs of the different 6MP PK models. RMSEs and MAEs of the patient trajectories computed with the individual patient parameters are calculated patientwise, the reported values are the means and medians, standard deviation in brackets.

	PK_{mm}^{6MP}	$PK_{mm,pop}^{6MP}$	PK_{lin}^{6MP}	$PK_{mm,pop,ae}^{6MP}$	$PK_{mm,pop,pe}^{6MP}$	$PK_{mm,pop,eps}^{6MP}$
**OBJ**	-5,574	-5,572	-4,960	-4,626	-5,286	-5,325
**Errors of the individual predictions**						
Median of RMSEs	0.21 (0.16)	0.21 (0.16)	0.23 (0.18)	0.21 (0.15)	0.21 (0.16)	0.21 (0.16)
Mean of RMSEs	0.24 (0.16)	0.24 (0.16)	0.26 (0.18)	0.24 (0.15)	0.24 (0.16)	0.24 (0.16)
Median of MAEs	0.17 (0.13)	0.17 (0.13)	0.17 (0.14)	0.16 (0.12)	0.16 (0.13)	0.17 (0.13)
Mean of MAEs	0.19 (0.13)	0.19 (0.13)	0.20 (0.14)	0.18 (0.12)	0.19 (0.13)	0.19 (0.13)

We compared linear and Michaelis-Menten kinetics for the 6MP PK model with both leading to similar RMSEs and MAEs of the individual predictions and the parameter estimation with Michaelis-Menten kinetics again leading to a lower objective function with a value of -5,574 than the one with linear kinetics with an objective function value of -4,960 (cf. Supplementary Table S6 online).

Similar to the MTX PK model with Michaelis-Menten kinetics, model PK_{mm}^{6MP} led to a 95%-CI for the coefficient of variation of K_{mm}^{6MP} that covers 0. We thus again estimated the model with the interindividual variability for K_{mm}^{6MP} removed. As Supplementary Table S6 online shows, model $PK_{mm,pop}^{6MP}$ led to a similar objective function

value, and the same RMSEs and MAEs of the individual predictions as model PK_{mm}^{6MP}, without any 95%-CI of the coefficients of variation covering 0.

In the next step, we compared the results of the cross-validation of both models $PK_{mm,pop}^{6MP}$ and PK_{lin}^{6MP}. The results of the latter one are presented in Supplementary Table S7 online, comparing the parameter values and the RMSEs and MAEs of the individual predictions of both the estimation using the whole data set and with the cross-validation set, showing again an expected increase in the RMSEs and MAEs, and almost no changes in the resulting parameters. In contrast to the MTX PK model with linear kinetics, this model leads to significant p-values < 0.05, rejecting the hypothesis that the true mean of the η-estimates is 0. Supplementary Figure S13 online plots the individual patient parameters of model PK_{lin}^{6MP} of the estimation using the whole data set against the cross-validation, showing only minor differences between the two.

*Supplementary Table S7: Parameter values of the model PK_{lin}^{6MP} estimated with the whole and the cross-validation data set, relative standard error (RSE) of fixed effect parameters and residual unexplained variability in brackets. RMSEs and MAEs of the patient trajectories computed with the individual patient parameters are each calculated for the whole data set with the standard deviation in brackets. Coefficients of variation (CV) are calculated as $\sqrt{exp(\omega^2)-1}$ with ω^2 as the variance of the interindividual variability estimated by NONMEM, and the 95%-CI being calculated accordingly. *Significant on the 0.05-level.*

	PK_{lin}^{6MP}	PK_{lin}^{6MP}, cross-validation
**Fixed effects parameters with RSE in %**		
K_{in}^{6MP} [1/day]	0.11 (12)	0.12 (14)
K_{eff}^{6MP} [1/day]	0.017 (12)	0.018 (14)
**CV with 95%-CI, η-Shrinkage in % and p-values**		
K_{in}^{6MP}	75 [64, 85], 22, 0.041*	61 [50, 70], 31, 0.028*
K_{eff}^{6MP}	61 [51, 70], 33, 0.023*	46 [37, 55], 45, 0.0088*
**Residual unexplained variability**		
Additive residual error	0.021 (9)	0.019 (12)
Proportional residual error	0.079 (4)	0.063 (6)
**Errors of the individual predictions**		
Median of RMSEs	0.23 (0.18)	0.30 (0.29)
Mean of RMSEs	0.26 (0.18)	0.36 (0.29)
Median of MAEs	0.17 (0.14)	0.23 (0.23)
Mean of MAEs	0.20 (0.14)	0.28 (0.23)

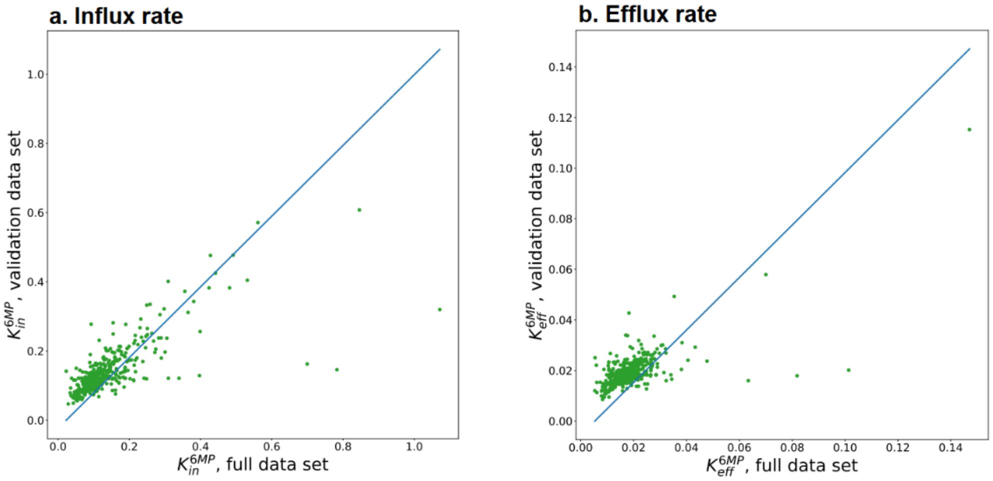

Supplementary Figure S13: Scatterplots of the individual patient parameters K_{in}^{6MP} and K_{eff}^{6MP} for the PK_{lin}^{6MP} model, parameters estimated with the full data set on the horizontal axis and with the validation data set on the vertical axis.

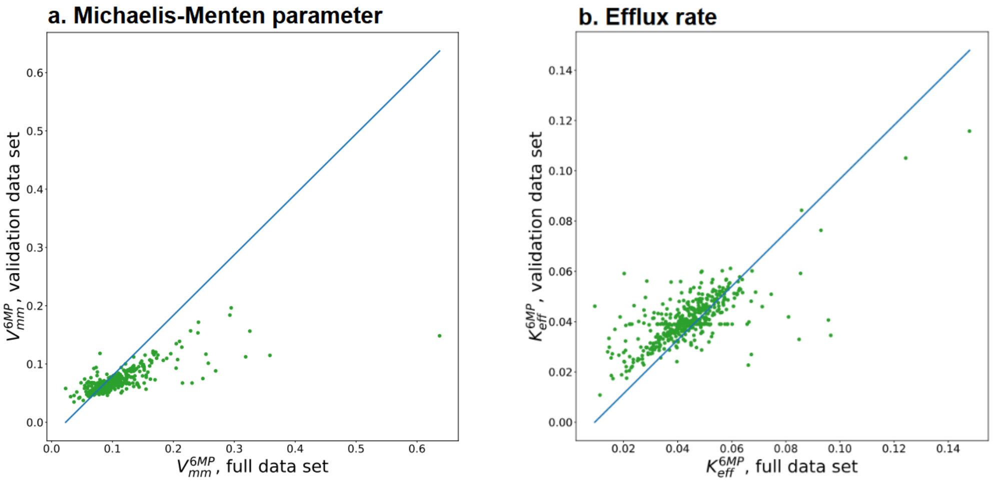

Supplementary Figure S14: Scatterplots of the individual patient parameters V_{mm}^{6MP} and K_{eff}^{6MP} for the model $PK_{mm,pop}^{6MP}$, parameters estimated with the full data set on the horizontal axis and with the validation data set on the vertical axis.

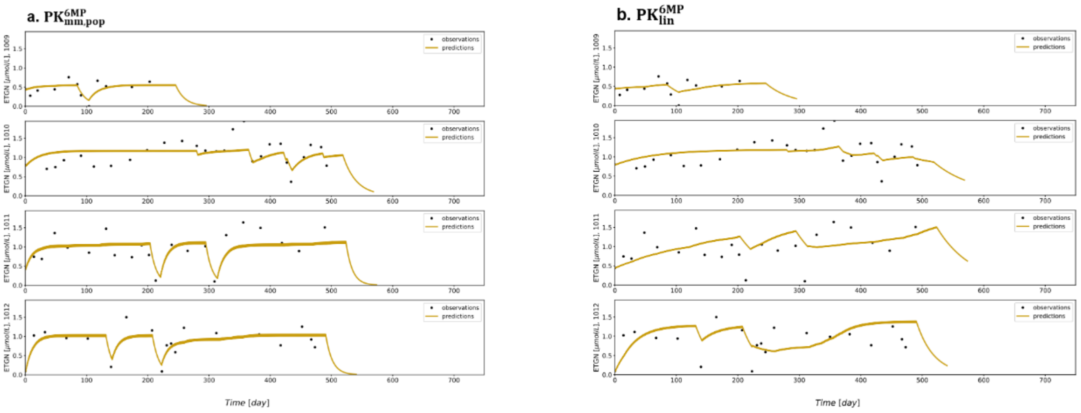

Supplementary Figure S15: Individual trajectories of four patients.

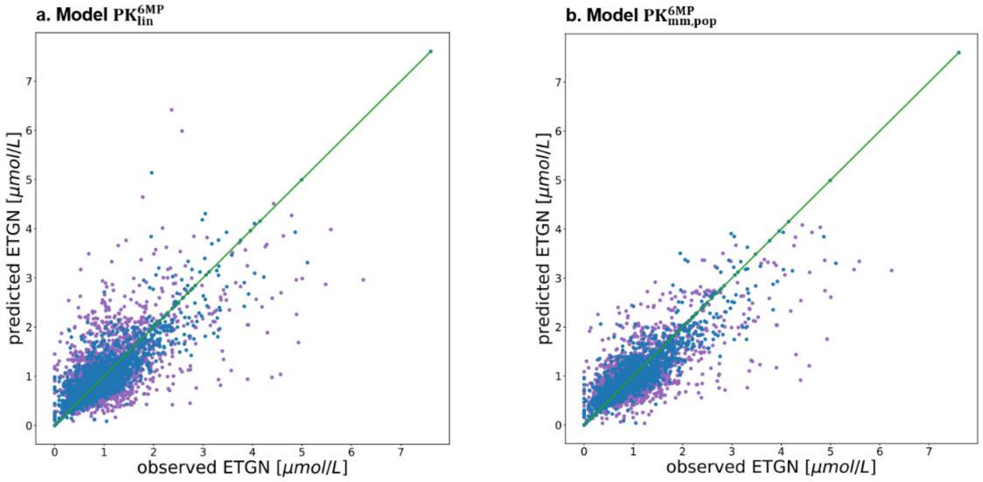

Supplementary Figure S16: Goodness of fit plots of observed and predicted E-TGN values. Estimation was done with the cross-validation data set, where blue dots are in-sample and purple dots are out-of-sample observations.

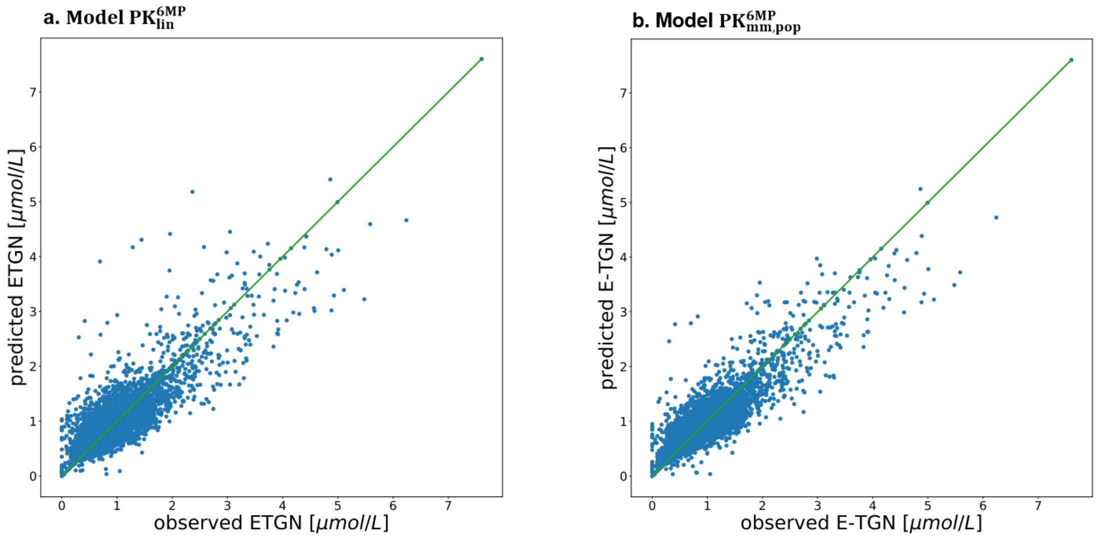

Supplementary Figure S17: Goodness-of-fit plots of observed and by the model PK_{lin}^{6MP} and model $PK_{mm,pop}^{6MP}$ predicted E-TGN values, estimation using the whole data set.

In contrast to the MTX PK models, the trajectories resulting from model $PK_{mm,pop}^{6MP}$ and PK_{lin}^{6MP} do not co-move as much (cf. Supplementary Figure S15 online), indicating that the linear kinetics might not be able to capture the dynamics of 6MP and that concentrations are reached where Michaelis-Menten kinetics are necessary to display the correct behavior.

The observations and predictions of both model variants PK_{mm}^{6MP} and PK_{lin}^{6MP} for the cross-validation are plotted against each other in Supplementary Figure S16 online, the ones for the estimation using the whole data set in Supplementary Figure S17 online, with those of the model with Michaelis-Menten kinetics showing a slightly better agreement of observed with predicted E-TGN values. Supplementary Figure S19 online displays the visual predictive checks for model $PK_{mm,pop}^{6MP}$ with 1000 simulations each using the final parameter values. Again, model $PK_{mm,pop}^{6MP}$ led to better results, especially for the 2.5th and 97.5th percentiles (cf. also Supplementary Figure S18 online for the visual predictive check of the model PK_{lin}^{6MP}).

We then tested the model $PK_{mm,pop}^{6MP}$ again against the same four variations as with the MTX PK model: we replaced the additive-proportional residual error model with a purely additive ($PK_{mm,pop,ae}^{6MP}$) and a purely proportional one ($PK_{mm,pop,pe}^{6MP}$), and we exchanged the initialization of the E-TGN compartment by an observation with either a direct estimation of the value, or the possibility of varying similarly to the residual error model ($PK_{mm,pop,eps}^{6MP}$). The model variant with the initial value estimated again led to a result with implausible parameter values and NONMEM not being able to estimate the covariance matrix and is thus not included here. All three other models resulted in a higher objective function value than $PK_{mm,pop}^{6MP}$, cf. Supplementary Table S6 online.

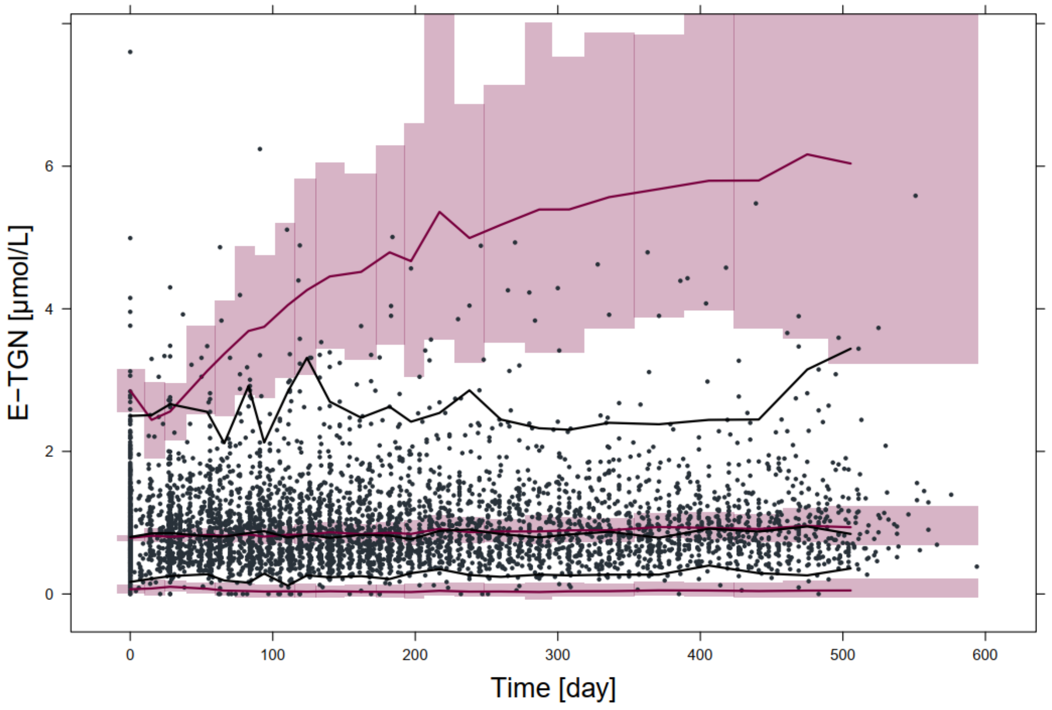

Supplementary Figure S18: Visual predictive check with 1000 simulations of the model PK_{lin}^{6MP}. The grey dots represent the observed E-TGN values, the black lines the corresponding median, 2.5th and 97.5th percentiles. The red lines show the median, 2.5th and 97.5th percentiles of the model predictions, the shaded areas represent the corresponding 95% confidence intervals.

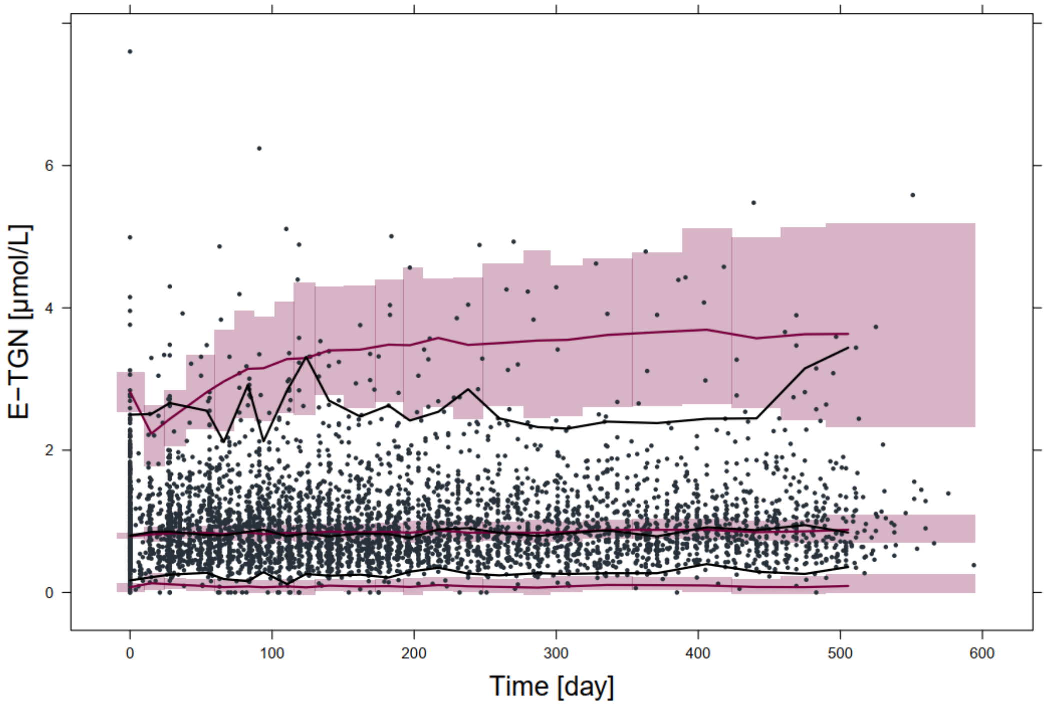

Supplementary Figure S19: Visual predictive check with 1000 simulations of the model $PK_{mm,pop}^{6MP}$. The grey dots represent the observed E-TGN values, the black lines the corresponding median, 2.5th and 97.5th percentiles. The red lines show the median, 2.5th and 97.5th percentiles of the model predictions, the shaded areas represent the corresponding 95% confidence intervals.

G Detailed Results for the full PKPD model

*Supplementary Table S8: Objective function values, RMSEs and MAEs and patient-wise minima, medians and maxima of observations and individual predictions of the different PKPD models. RMSEs and MAEs of the patient trajectories computed with the individual patient parameters are calculated patientwise, the reported values are the means and medians. Standard deviation in brackets. The objective function values of the PKPD models are obtained by 15 final IMP iterations that still show fluctuations, hence, the mean and standard deviation of these iterations are reported here. *The objective function value of the model $PKPD^{Jost}$ cannot be compared to those of the other models, since the parameter estimation only depends on the ANC values.*

	$PKPD^{Jost}$	$PKPD^{6MP}_{lin}$	$PKPD^{6MP}_{lin,mm}$	$PKPD^{6MP,MTX}_{lin}$	$PKPD^{6MP,MTX}_{lin,mm}$
**OBJ**	11,251 (53)*	-31,114 (81)	-31,531 (61)	-31,076 (92)	-31,479 (71)
**ANC errors of the individual predictions**					
Median of RMSEs	0.85 (0.52)	0.87 (0.52)	0.92 (0.53)	0.87 (0.53)	0.89 (0.52)
Mean of RMSEs	0.97 (0.52)	0.97 (0.52)	1.01 (0.53)	0.97 (0.53)	0.99 (0.52)
Median of MAEs	0.65 (0.39)	0.67 (0.39)	0.71 (0.39)	0.67 (0.40)	0.68 (0.38)
Mean of MAEs	0.75 (0.39)	0.75 (0.39)	0.78 (0.39)	0.76 (0.40)	0.76 (0.38)
**E-MTX errors of the individual predictions**					
Median of RMSEs	-	0.0042 (0.0028)	0.0042 (0.0028)	0.0042 (0.0028)	0.0042 (0.0028)
Mean of RMSEs	-	0.0047 (0.0028)	0.0047 (0.0028)	0.0047 (0.0028)	0.0047 (0.0028)
Median of MAEs	-	0.0033 (0.0022)	0.0033 (0.0022)	0.0033 (0.0022)	0.0033 (0.0022)
Mean of MAEs	-	0.0036 (0.0022)	0.0036 (0.0022)	0.0036 (0.0022)	0.0036 (0.0022)
**E-TGN errors of the individual predictions**					
Median of RMSEs	-	0.22 (0.17)	0.21 (0.16)	0.22 (0.17)	0.20 (0.16)
Mean of RMSEs	-	0.25 (0.17)	0.24 (0.16)	0.25 (0.17)	0.24 (0.16)
Median of MAEs	-	0.17 (0.14)	0.16 (0.13)	0.17 (0.14)	0.16 (0.13)
Mean of MAEs	-	0.19 (0.14)	0.19 (0.13)	0.19 (0.14)	0.18 (0.13)
**Medians of the patientwise minima, medians and maxima of the observations and the individual predictions**					
Minima of obs	0.50 (0.49)	0.50 (0.49)	0.50 (0.49)	0.50 (0.49)	0.50 (0.49)
Minima of ipreds	1.14 (0.43)	1.16 (0.46)	1.23 (0.45)	1.16 (0.45)	1.18 (0.46)
Median of obs	1.63 (0.60)	1.63 (0.60)	1.63 (0.60)	1.63 (0.60)	1.63 (0.60)
Median of ipreds	1.73 (0.60)	1.76 (0.60)	1.78 (0.62)	1.76 (0.59)	1.77 (0.59)
Maxima of obs	4.4 (2.59)	4.4 (2.59)	4.4 (2.59)	4.4 (2.59)	4.4 (2.59)
Maxima of ipreds	2.76 (1.53)	2.73 (1.45)	2.58 (1.25)	2.78 (1.42)	2.64 (1.32)

To be able to compare the objective function values of the different models for the model evaluation, we estimated the parameters for both PK submodels for all PKPD models, irrespective of them both being included in the effect function. The RMSEs and MAEs of the individual predictions show only negligible differences, with the RMSEs and MAEs of the PK submodels not differing from the results when estimating only the corresponding PK models.

The objective function values are comparable for both models with only linear kinetics in the PK submodels ($PKPD^{6MP}_{lin}$ and $PKPD^{6MP,MTX}_{lin}$), and for both models with linear kinetics in the MTX PK model and Michaelis-Menten kinetics in the 6MP PK submodel ($PKPD^{6MP}_{lin,mm}$ and $PKPD^{6MP,MTX}_{lin,mm}$), hence including E-MTX in the effect function does not lead to better results than basing the effect function only on E-TGN. Overall, the models with Michaelis-Menten kinetics in the 6MP PK submodel led to lower objective function values than the two models with purely linear PK kinetics. Comparing all models with regard to their ability to capture the full range of observations (with a median of patientwise minima of 0.5 G/L and a median of patientwise maxima of 4.4 G/L) reveals similar results: all models are able to predict the median ANC values well, but display a smaller range for the individual predictions than the observations show, leading to higher patientwise minima (with a median of patientwise minima of 1.14-1.23 G/L) and lower patientwise maxima (with a median of patient-wise maxima of 2.58-2.78 G/L, cf. Supplementary Table S8 online).

The cross-validation of the model $PKPD_{lin}^{6MP}$ led to an immense increase in RMSEs and MAEs of the individual predictions, with mean($RMSE_i$) = 13720 and mean(MAE_i) = 3804. Having a closer look at the individual patients revealed an implausible increase in ANC values for one patient with an estimated individual parameter $K_{in,i}^{6MP}$ = 1.02 which was more than three times the size of the population parameter K_{in}^{6MP} = 0.27, but still in agreement with the model results. The treatment schedule of this patient showed that in the cross-validation data set, the highest 6MP dose of this patient was not included and led to a strong increase in E-TGN values and therefore a severe decrease in ANC values, which then induced the sharp increase in ANC values due to the feedback mechanism (cf. Supplementary Figure S22 online). We therefore concluded that for 6MP, the PK model with linear kinetics is not able to capture the dynamics of 6MP treatment well enough for all plausible treatment schedules and excluded both $PKPD_{lin}^{6MP}$ and $PKPD_{lin}^{6MP,MTX}$ model from further analysis.

Supplementary Table S9: RMSEs and MAEs of the individual predictions computed with the individual patient parameters of the cross-validation of models $PKPD_{lin,mm}^{6MP}$ and $PKPD_{lin,mm}^{6MP,MTX}$.

	$\boldsymbol{PKPD_{lin,mm}^{6MP}}$	$\boldsymbol{PKPD_{lin,mm}^{6MP,MTX}}$
**E-TGN errors of the individual predictions of the cross-validation**		
Median of RMSEs	0.25 (0.23)	0.25 (0.23)
Mean of RMSEs	0.30 (0.23)	0.30 (0.23)
Median of MAEs	0.19 (0.18)	0.19 (0.18)
Mean of MAEs	0.23 (0.18)	0.23 (0.18)
**E-MTX errors of the individual predictions of the cross-validation**		
Median of RMSEs	0.0051 (0.0046)	0.0051 (0.0046)
Mean of RMSEs	0.0062 (0.0046)	0.0062 (0.0046)
Median of MAEs	0.0040 (0.0036)	0.0040 (0.0026)
Mean of MAEs	0.0047 (0.0036)	0.0047 (0.0036)

In Table Supplementary S10 online, the results of the parameter estimation using the whole data set and for the cross-validation for the model $PKPD_{lin,mm}^{6MP,MTX}$ can be found. Similar to model $PKPD_{lin,mm}^{6MP}$, the fixed effect parameter K_{mm}^{6MP} still decreases, but again not as sharply as when estimating only the 6MP PK model. The same applies to its relative standard error, which does not show the immense increase for the cross-validation of the full PKPD model. The coefficients of variation show only negligible changes for the cross-validation. Compared to model $PKPD_{lin,mm}^{6MP}$, the parameter estimation of the full PKPD model leads to similar results as those of the estimation of only the PK submodels (cf. Tables 2 and 4), except for the 6MP PK submodel, where again both V_{mm}^{6MP} and K_{mm}^{6MP} increase distinctly. Again, integrating E-MTX into the effect function does not improve the model.

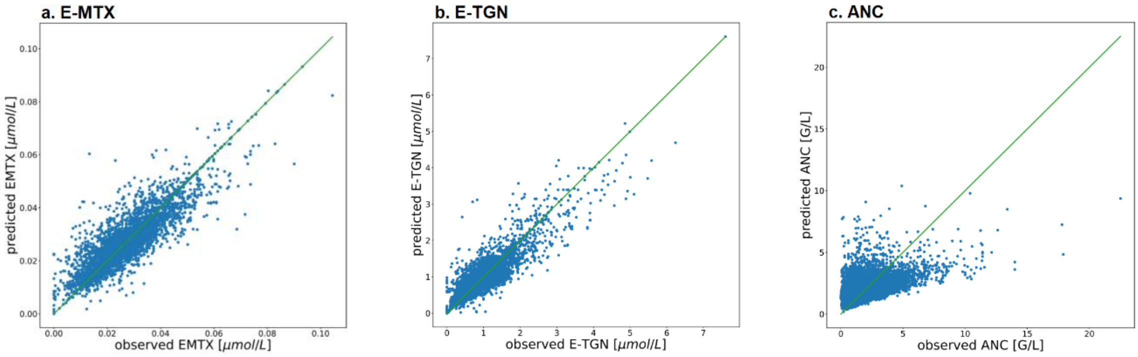

Supplementary Figure S20: Goodness-of-fit plots of observed and by the model $PKPD_{lin,mm}^{6MP}$ predicted E-MTX, E-TGN and ANC values, estimation using the whole data set.

Supplementary Table S10: Parameter values of the model $PKPD_{lin,mm}^{6MP,MTX}$ estimated with the whole and the cross-validation data set, relative standard error (RSE) of fixed effect parameters and residual unexplained variability in brackets. Coefficients of variation are calculated as $\sqrt{exp(\omega^2) - 1}$ with ω^2 as as the variance of the interindividual variability estimated by NONMEM, and the 95%-CI being calculated accordingly.

	$PKPD_{lin,mm}^{6MP,MTX}$		$PKPD_{lin,mm}^{6MP,MTX}$, cross-validation	
	**Fixed effect parameters with RSE in %**	**CV with 95%-CI, η-Shrinkage in % and p-values**	**Fixed effect parameters with RSE in %**	**CV with 95%-CI, η-Shrinkage in % and p-values**
K_{in}^{MTX} [1/day]	0.031 (1)	34 [29, 40], 29, 0.86	0.027 (1)	37 [32, 42], 33, 0.98
K_{eff}^{MTX} [1/day]	0.018 (1)	32 [20, 41], 32, 0.97	0.017 (1)	27 [21, 32], 45, 0.81
V_{mm}^{6MP} [µmol/L/day]	0.26 (5)	54 [28, 74], 27, 0.68	0.16 (4)	42 [34, 49], 37, 0.99
K_{mm}^{6MP} [µmol/L]	0.20 (0.02)	-	0.079 ($2.2 \cdot 10^{-9}$)	-
K_{eff}^{6MP} [1/day]	0.057 (2)	54 [36, 69], 28, 0.77	0.046 (2)	46 [39, 52], 32, 0.97
$base$[G/L]	2.5 (3)	28 [24, 31], 25, 0.91	2.2 (4)	28 [24, 32], 29, 0.99
k_{tr} [1/day]	0.16 (3)	79 [64, 94], 37, 0.35	0.17 (3)	71 [59, 82], 45, 0.21
$slope^{6MP}$ [L/µmol]	0.13 (4)	97 [78, 117], 41, 0.99	0.12 (6)	97 [72, 121], 49, 0.78
γ	0.77 (4)	11 [9, 12], 47, 0.44	0.82 (11)	14 [7, 19], 57, 0.85
$inieff$	0.75 (12)	52 [46, 59], 22, 0.89	0.84 (21)	48 [44, 52], 27, 0.90
$slope^{MTX}$ [L/µmol]	2.5 (14)	91 [66, 115], 59, 0.43	1.5 (35)	137 [127, 148], 63, 0.98
**Residual unexplained variability**				
Additive, MTX	0.000018 (7)		0.000021 (8)	
Proportional, MTX	0.024 (8)		0.0080 (23)	
Additive, 6MP	0.026 (16)		0.019 (11)	
Proportional, 6MP	0.059 (7)		0.051 (6)	
Proportional, ANC	0.25 (2)		0.22 (3)	

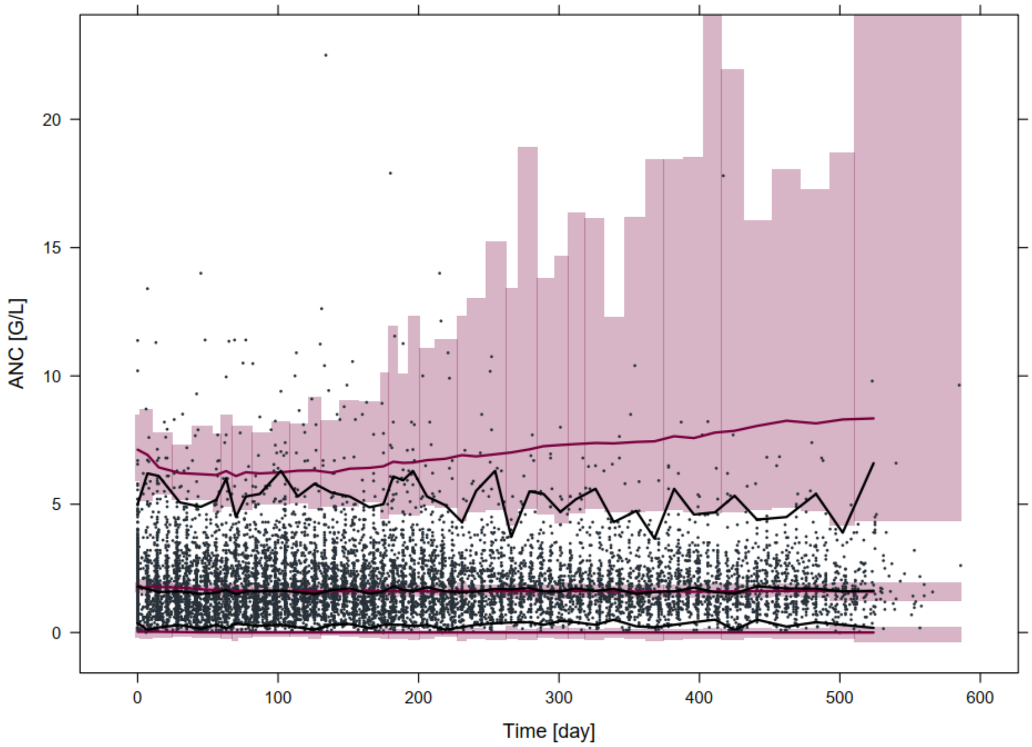

Supplementary Figure S21: Visual predictive check with 1000 simulations of the final model $PKPD^{6MP}_{lin,mm}$. The grey dots represent the observed ANC values, the black lines the corresponding median, 2.5th and 97.5th percentiles. The red lines show the median, 2.5th and 97.5th percentiles of the model predictions, the shaded areas represent the corresponding 95% confidence intervals.

H Cross-validation of model $PKPD^{6MP}_{lin}$

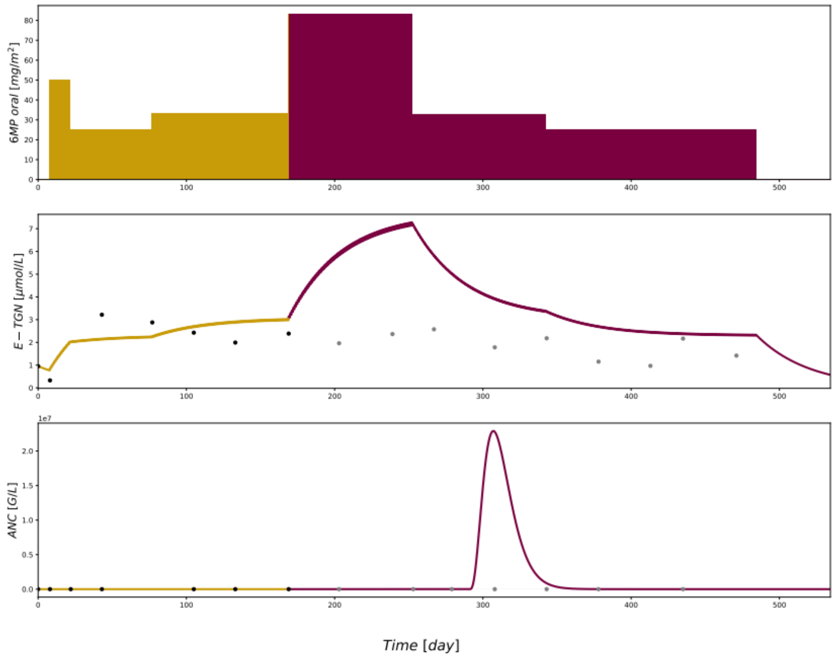

Supplementary Figure S22: Patient trajectory predicted by model $PKPD^{6MP}_{lin}$, in yellow and black the time span in the cross-validation data set, in red and grey the out-of-sample observations and predictions.

I Final PKPD model

The final PKPD model consists of the ODE system below:

$$\frac{dX_{GI}^{6MP}}{dt} = -k_a^{6MP} \cdot X_{GI}^{6MP}$$

$$\frac{dX_{GI}^{6MP}}{dt} = k_a^{6MP} \cdot X_{GI}^{6MP} - k_e^{6MP} \cdot X_C^{6MP}$$

$$\frac{dX_E^{6MP}}{dt} = \frac{V_{mm}^{6MP} \cdot X_C^{6MP} / V_C^{6MP}}{K_{mm}^{6MP} + X_C^{6MP} / V_C^{6MP}} - K_{eff}^{6MP} \cdot X_E^{6MP}$$

$$\frac{dX_{prol}}{dt} = k_{tr} \cdot X_{prol} \cdot \left(1 - E_{drug}\right) \cdot fb - k_{tr} \cdot X_{prol}$$

$$\frac{dX_{tr1}}{dt} = k_{tr} \cdot (X_{prol} - X_{tr1})$$

$$\frac{dX_{tr2}}{dt} = k_{tr} \cdot (X_{tr1} - X_{tr2})$$

$$\frac{dX_{tr3}}{dt} = k_{tr} \cdot (X_{tr2} - X_{tr3})$$

$$\frac{dX_{ma}}{dt} = k_{tr} \cdot X_{tr3} - k_{circ} \cdot X_{ma}$$

with

$$fb = \left(\frac{base}{X_{ma}}\right)^{\gamma}$$

$$E_{drug} = slope^{6MP} \cdot X_E^{6MP}$$

The compartments were initialized in the following way:

$$X_{GI}^{6MP}(0) = 0$$

$$X_C^{6MP}(0) = 0$$

$$X_E^{6MP}(0) = INITGN$$

$$X_{prol}(0) = inieff \cdot base \cdot \frac{k_{ma}}{k_{tr}}$$

$$X_{tr1}(0) = inieff \cdot base \cdot \frac{k_{ma}}{k_{tr}}$$

$$X_{tr2}(0) = inieff \cdot base \cdot \frac{k_{ma}}{k_{tr}}$$

$$X_{tr3}(0) = inieff \cdot base \cdot \frac{k_{ma}}{k_{tr}}$$

$$X_{ma}(0) = inieff \cdot base$$

Supplementary Table S11 shows the values of the fixed parameters of the final PKPD model.

Supplementary Table S11: Fixed parameters of the final PKPD model.

**Submodel**	**Parameter**	**Value**	**Source**
$PKPD^{6MP}$	Bioavailability F^{6MP}	0.12	Brunton et al. (2005)
	Absorption rate k_a^{6MP}	21.07 1/day	Lennard et al. (1986)
	Elimination rate k_e^{6MP}	15.4 1/day	Lennard et al. (1986)
	Central volume V_C^{6MP}	20.1911 L/m^2	Lennard et al. (1986)
$PKPD$	k_{circ}	2.3765 1/day	Jost et al. (2020)

J Exemplary individual patient trajectories

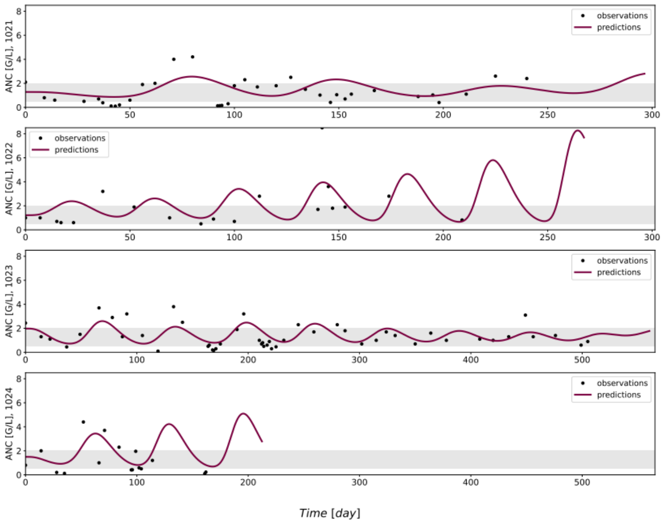

Supplementary Figure S23: Individual ANC trajectories of four patients predicted by the final PKPD model.

Four patients exhibiting qualitatively different oscillating behavior were selected after a visual check of patient ANC trajectories.

K Sensitivity Analysis

Supplementary Table S12: Sensitivity analysis of the fixed parameters of the final PKPD.

**Submodel**	**Parameter**	**Value**	**Maximal distance to reference ANC trajectory**	**Median and interquartile range of the trajectory with the maximal distance to the reference ANC trajectory**
$PKPD^{6MP}$	Bioavailability F^{6MP}	0.12	0.12	0.082 (0.047)
	Absorption rate k_a^{6MP}	21.07 1/day	0.49	0.34 (0.17)
	Elimination rate k_e^{6MP}	15.4 1/day	0.093	0.063 (0.035)
	Central volume V_C^{6MP}	20.1911 L/m^2	0.11	0.071 (0.039)
$PKPD$	k_{circ}	2.3765 1/day	0.0013	0.00048 (0.00062)

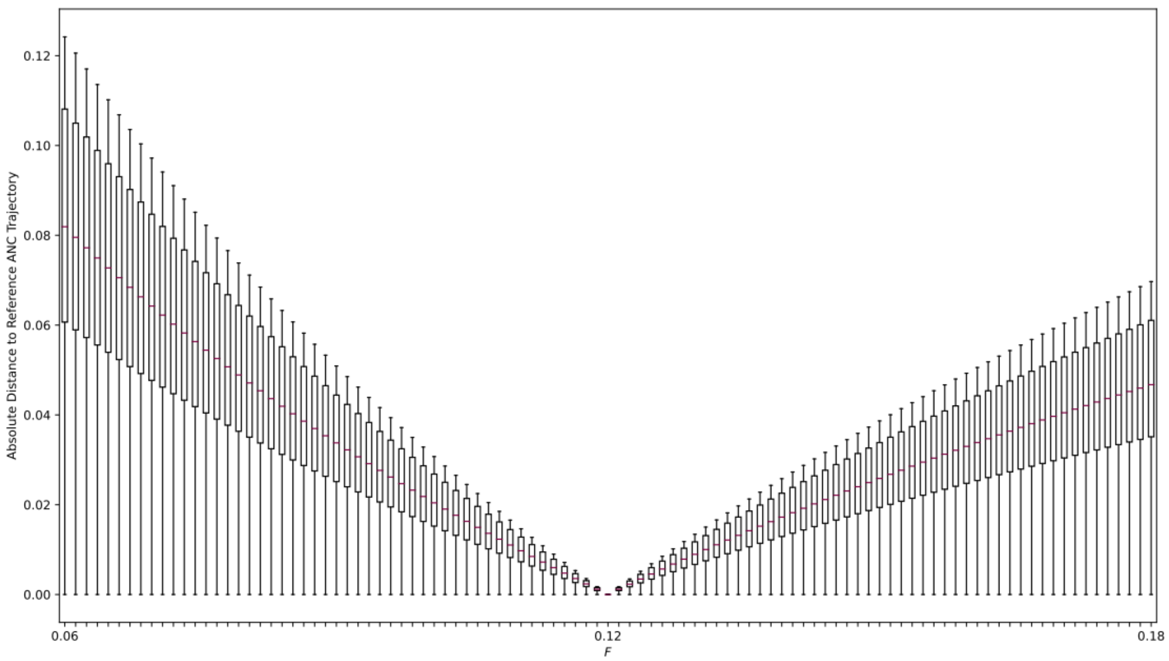

Supplementary Figure S24: Absolute distance to the reference ANC trajectory of the final PKPD model depending on the bioavailability F. 100 Simulations with F equally spaced in [0.06, 0.18] and estimated parameters fixed to population parameters. F = 0.12 for the reference trajectory. Each boxplot shows the median, the quartiles and the range of the absolute distance to the reference trajectory.

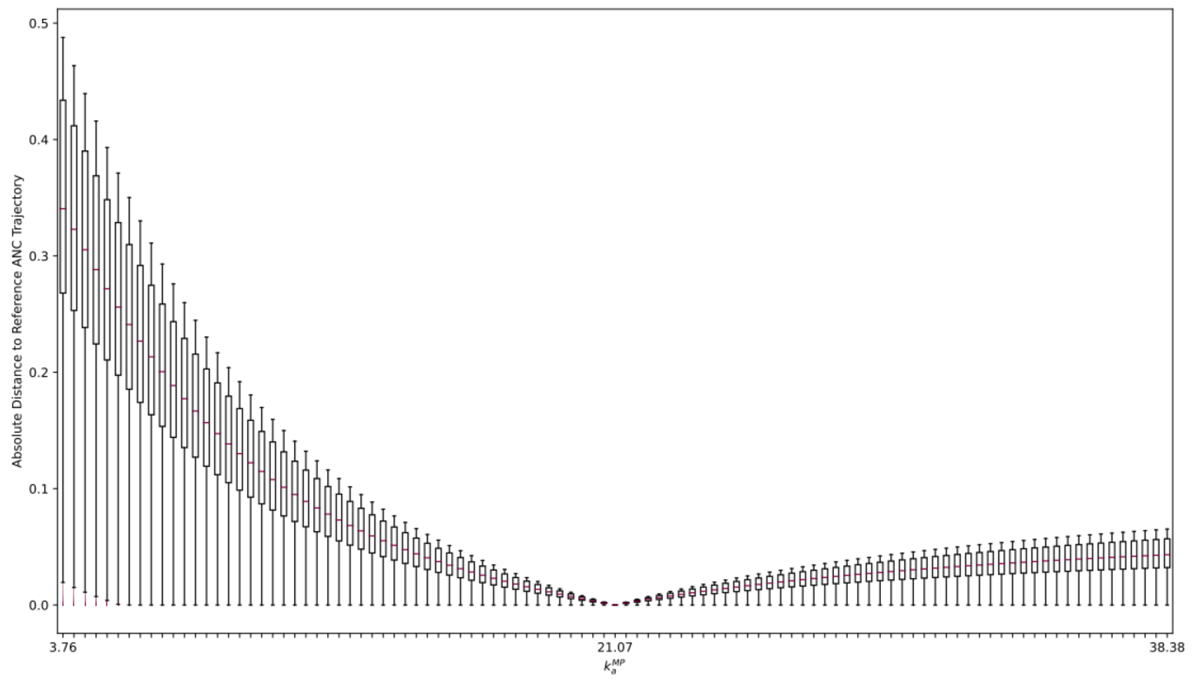

Supplementary Figure S25: Absolute distance to the reference ANC trajectory of the final PKPD model depending on the absorption rate k_a^{6MP}. 100 Simulations with k_a^{6MP} equally spaced in [3.76, 38.38] and estimated parameters fixed to population parameters. k_a^{6MP} = 21.07 for the reference trajectory. Each boxplot shows the median, the quartiles, the range and outliers of the absolute distance to the reference trajectory.

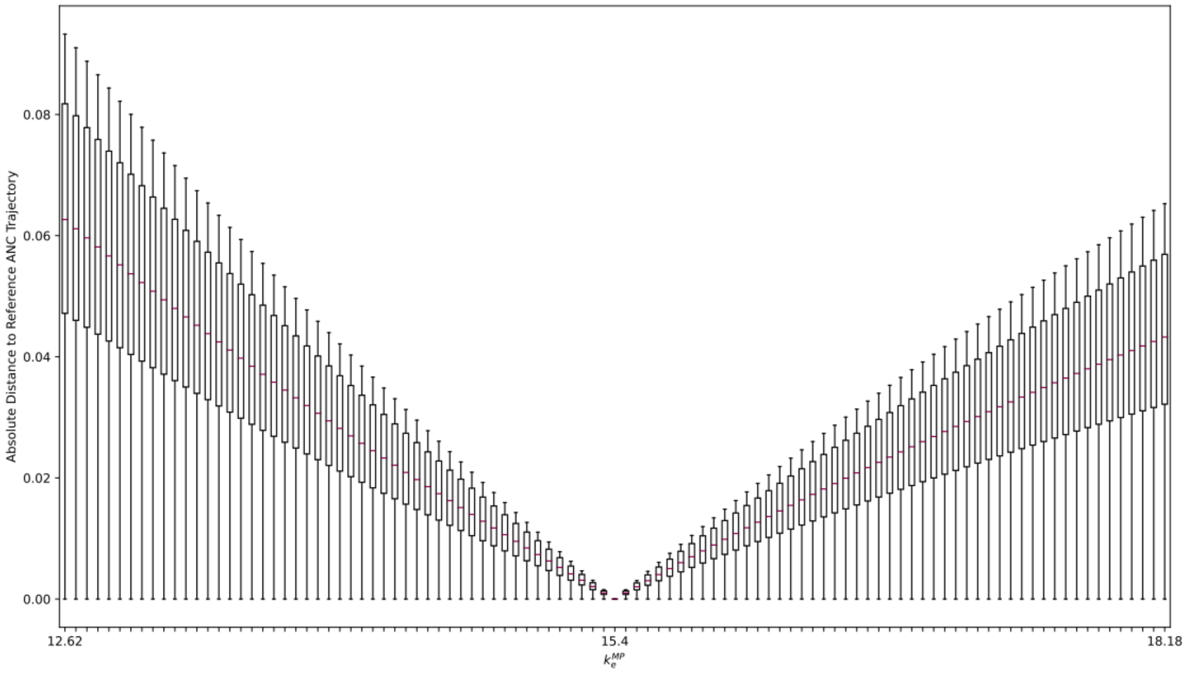

Supplementary Figure S26: Absolute distance to the reference ANC trajectory of the final PKPD model depending on the elimination rate k_e^{6MP}. 100 Simulations with k_e^{6MP} equally spaced in [12.62, 18.18] and estimated parameters fixed to population parameters. k_e^{6MP} = 15.40 for the reference trajectory. Each boxplot shows the median, the quartiles and the range of the absolute distance to the reference trajectory.

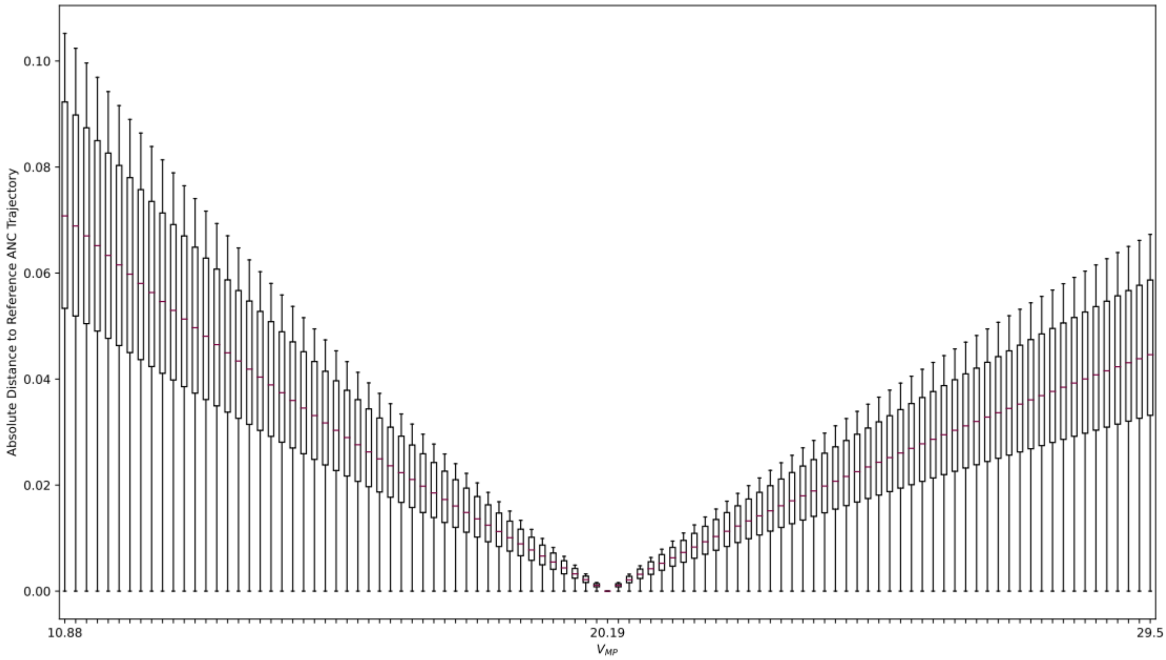

Supplementary Figure S27: Absolute distance to the reference ANC trajectory of the final PKPD model depending on the central volume V_C^{6MP} 100 Simulations with V_C^{6MP} equally spaced in [10.88, 29.50] and estimated parameters fixed to population parameters. V_C^{6MP} = 20.1911 for the reference trajectory. Each boxplot shows the median, the quartiles and the range of the absolute distance to the reference trajectory.

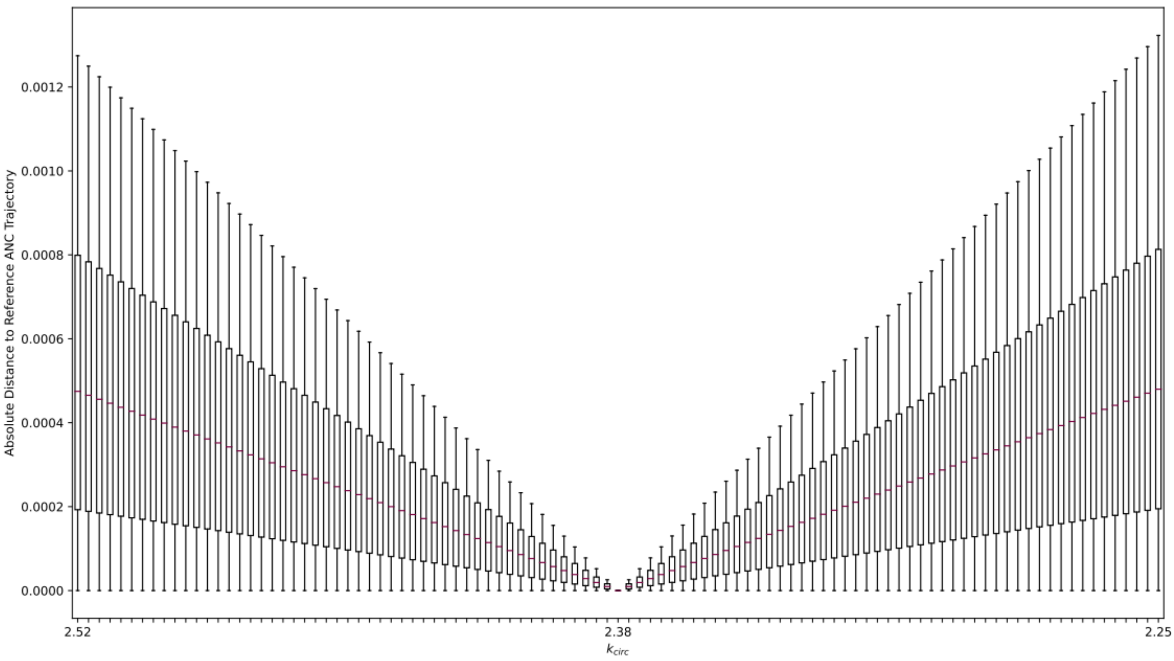

Supplementary Figure S28: Absolute distance to the reference ANC trajectory of the final PKPD model depending on the death rate of mature neutrophils k_{circ}. 100 Simulations with $t_{0.5}$ equally spaced in [0.28, 0.31], k_{circ} = ln(2)/$t_{0.5}$ therefore in [0.06, 0.18] and estimated parameters fixed to population parameters. k_{circ} = 2.3765 for the reference trajectory. Each boxplot shows the median, the quartiles and the range of the absolute distance to the reference trajectory.
